# Intrinsic disorder in the regulatory N-terminal domain of diacylglycerol acyltransferase 1 from *Brassica napus*

**DOI:** 10.1038/s41598-018-34339-1

**Published:** 2018-11-12

**Authors:** Rashmi Panigrahi, Tsutomu Matsui, Andrew H. Song, Kristian Mark P. Caldo, Howard S. Young, Randall J. Weselake, M. Joanne Lemieux

**Affiliations:** 1 Department of Biochemistry, University of Alberta, Edmonton, Alberta T6G 2H7 Canada; 2 Stanford Synchrotron Radiation Lightsource, SLAC National Accelerator Laboratory, Stanford University, Menlo Park, CA 94025 USA; 3 Department of Agricultural, Food and Nutritional Science, University of Alberta, Edmonton, Alberta T6G 2P5 Canada

## Abstract

Proteins with multifunctional regulatory domains often demonstrate structural plasticity or protein disorder, allowing the binding of multiple regulatory factors and post-translational modifications. While the importance of protein disorder is clear, it also poses a challenge for *in vitro* characterization. Here, we report protein intrinsic disorder in a plant molecular system, which despite its prevalence is less studied. We present a detailed biophysical characterization of the entire cytoplasmic N-terminal domain of *Brassica napus* diacylglycerol acyltransferase, (DGAT1), which includes an inhibitory module and allosteric binding sites. Our results demonstrate that the monomeric N-terminal domain can be stabilized for biophysical characterization and is largely intrinsically disordered in solution. This domain interacts with allosteric modulators of DGAT1, CoA and oleoyl-CoA, at micromolar concentrations. While solution scattering studies indicate conformational heterogeneity in the N-terminal domain of DGAT1, there is a small gain of secondary structure induced by ligand binding.

## Introduction

The classic protein structure and function paradigm reveals three-dimensional structures of proteins with precisely positioned functional groups that facilitate interactions with substrates or protein partners. However, numerous studies over the past few decades have demonstrated the functional significance of protein disordered regions as an exception to this general rule^1–3^, particularly in eukaryotic proteomes. Disordered proteins have been identified as a unique protein tribe^4^, which includes entire polypeptide chains referred to as “intrinsically disordered proteins (IDPs)” or localized domains of proteins referred to as “intrinsically disordered regions (IDRs)”. Interestingly, IDRs with over 40 amino acid residues are found in more than 33% of the sequenced eukaryotic proteins^5,6^. They differ from globular proteins in having a unique signature in a charge-hydropathy plot^7^. The disordered regions are enriched with charged residues along with low content of hydrophobic residues. Sequence analyses show that they are abundant in disorder-promoting residues (Ala, Arg, Gly, Gln, Ser, Glu, Lys, and Pro) and depleted of order-promoting counterparts (Trp, Tyr, Phe, Ile, Leu, Val, Cys, and Asn)^4,8–10^. Prediction of disordered regions in proteins is not only based on the charge-hydropathy correlation, but also on various other physico-chemical property scales such as helix propensity, strand propensity, and aromaticity^9^. IDRs/IDPs, for the most part, lack single ordered structure under physiological conditions (at least *in vitro*)^11^. Hence, they exist as heterogeneous ensembles of conformers where due to temporal fluctuations, a single set of backbone Ramachandran angles cannot be used to define their conformational properties. Thus, the IDPs/IDRs are known to have large hydrodynamic radii relative to folded proteins with a similar chain length. The lack of well-defined tertiary structure allows these disordered proteins to discriminate themselves from their folded counterparts, not only based on structure but also in terms of their function^12^.

The lack of rigid structure for IDRs/IDPs provides a broad functional advantage^1,6,13,14^, such as a larger interaction surface that can easily overcome steric hindrances, an ability to interact with structurally diverse partners, the ability to gain a specific conformation upon interaction with partners, the ability to stay substantially disordered in bound state, an efficient regulation through post-translational modifications, and the ability to be associated with interaction cascades. Therefore, IDRs are abundant in the proteins of higher order organisms associated with recognition, regulation and signaling^1,11,15–17^. Intrinsic disorder, being crucial for various biological functions, exists in various environments. The amino-acid compositions of IDPs/IDRs are specific for their local/global environment. For instance, in transmembrane (TM) proteins, TM segments are well structured due to their low dielectric constants within the lipid bilayer^18^, the exterior of these domains being apolar^19^. IDRs being highly charged and polar are expected to localize within regions external to the membrane^20^. Cytoplasmic domains of TM proteins, however, contain three-fold more disorder than their extracellular counterparts^21^. IDRs are also known to be potential drivers of membrane curvature, an event crucial for cellular physiology. Reports suggest that 50% of TM proteins have IDRs with more than 30 amino acid residues, and these are highly phosphorylated^22^.

Intrinsic disorder in plant proteins has been reported to be essential for the stress response^3^. The formation of a versatile complex network is effective for responding to change in unavoidable environmental stress. Recently, an IDR spanning the N-terminal cytosolic domain of an intramembrane enzyme, diacylglycerol acyltransferase1 (DGAT1) from canola-type *Brassica napus*, has been identified^23^. The IDR spans amino acid residues 1–80, while residues 81–113 have a folded structure. DGAT1 (EC 2.3.1.20) catalyzes the acyl-coenzyme A (CoA)-dependent acylation of *sn*-1, 2-diacylglycerol (DAG) to produce triacylglycerol (TAG) and CoA^24,25^. TAG serves as an energy source for germination in plants, a component of edible oil, and a petrochemical alternative. This enzyme has a substantial effect on carbon flux into seed oil and hence a molecular understanding of the regulatory mechanism of DGAT1 is essential for its genetic manipulation to increase seed oil production in oleaginous plants^24,26^.

**Figure 1:**
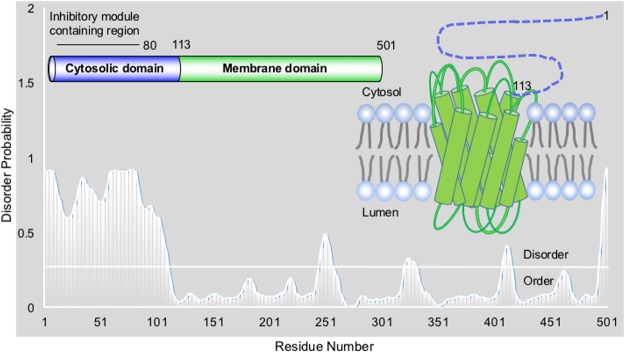
Disorder propensity representation for the full-length BnaDGAT1_1-501_ sequence (isoform BnaC.DGAT1) using DISOPRED. Disorder threshold is indicated by white line distinguishing between order and disorder. a). Inset 1: Cartoon representation of domains in BnaDGAT1; inset 2: A cartoon model of BnaDGAT1 embedded within the lipid bilayer, with the membrane domain depicted in green and the cytosolic domain depicted by a blue dashed line.

*B*. *napus* DGAT1 (BnaDGAT1) is a polytopic membrane protein localized on the endoplasmic reticulum (ER) with its N-terminal domain (residues 1–113) localized to the cytoplasm. (Fig. 1 inset). Currently no crystal structure of DGAT1 has been solved. Topological studies on the murine DGAT1 revealed the putative active site of murine DGAT1 is predicted to have binding sites for both acyl-CoA and DAG and a predicted ER lumen-facing enzyme active site^27^. The N-terminal region of BnaDGAT1 and a mammalian (murine) DGAT1 have been shown to interact with acyl-CoA^23,28,29^. Furthermore, crosslinking studies have shown that BnaDGAT1 has the ability to self-associate in membranes, but this is dependent upon the presence of the first 80 residues of the cytoplasmic N-terminal domain^23,28^. Various studies have suggested that plant DGAT1 enzymes are allosterically regulated^28–30^. In BnaDGAT1, acyl-CoA has been shown to act as both positive effector and acyl-donor whereas CoA is a negative effector. Both effectors have been shown to interact with the same non-catalytic site within the cytoplasmic N-terminal domain of the enzyme^23^. Lipidex-1000 binding assays have shown that CoA can displace acyl-CoA from this site^28^ and kinetic analysis has shown that CoA is a non-competitive inhibitor of the enzyme^23,28^. The acyl-CoA/CoA binding site has been shown to be located within amino acid residues 81–113. In contrast, an autoinhibitory domain within residues 1–80 has been identified; truncation of BnaDGAT1 to remove the first 80 amino acid residues resulted in an increase in enzyme activity^23^. Despite the growing biochemical evidence, there is lack of structural and biophysical understanding of the entire regulatory region of DGAT1 due to the disordered nature of this domain.

In this study, we investigated the interaction of CoA and oleoyl-CoA with the cytoplasmic N-terminal region (BnaDGAT1_1-113_) of isoform BnaC.DGAT1.a^23^ and a truncation, the autoinhibitory domain containing region (BnaDGAT1_1-80_), using isothermal titration calorimetry (ITC). Furthermore, we used circular dichroism (CD) and small-angle X-ray scattering (SAXS) to understand the conformational changes associated with interactions with these effectors. In addition, we provide biophysical evidence that the autoinhibitory region also interacts with CoA or acyl-CoA bringing about secondary structural changes. While both of these regions of BnaDGAT1 have the capacity to bind the above ligands, we show the entire region, amino acid residues 1–113, is needed for optimal binding and the secondary structural changes associated with this event.

## Results

### Bioinformatic analysis of the cytosolic domain DGAT1_1–113_

Our group recently reported an NMR structure for part of the BnaDGAT1 (isoform BnaC.DGAT1.a) N-terminal domain (residues 81–113)^23^; however, there are no publications describing the three-dimensional structure of the entire cytosolic domain (1–113) or the polypeptide beyond amino acid residue 113 which contains the multiple TM domains. DisoPred^31^ (Fig. 1), Globplot2^32^ and FoldIndex^33^ (Supplementary Figure 1A,B) analyses confirmed our previous observation that the N-terminal cytosolic domain (DGAT1_1-113_) is intrinsically disordered^23^. Although there are 14 predicted phosphorylation sites in DGAT1 from *Arabidopsis thaliana* (Supplementary Figure 1C), Ser31 and Ser36 in the N-terminal domain have been shown to have potential phosphorylation sites using mass spectrometry^34^. Furthermore, Gly7 is predicted using GPS-Lipid^35^ to be N-myristoylated. Myristate could potentially act as a hydrophobic anchor tethering the cytosolic domain to the phospholipid bilayer of the ER^14,36^. Given the potential for regulatory post-translational modifications in this region, experimental approaches are hence required to validate its intrinsic disorder.

### N-terminally tagged forms of BnaDGAT1_1–113_ and BnaDGAT_1–80_ are non-globular and monomeric *in vitro*

**Figure 2:**
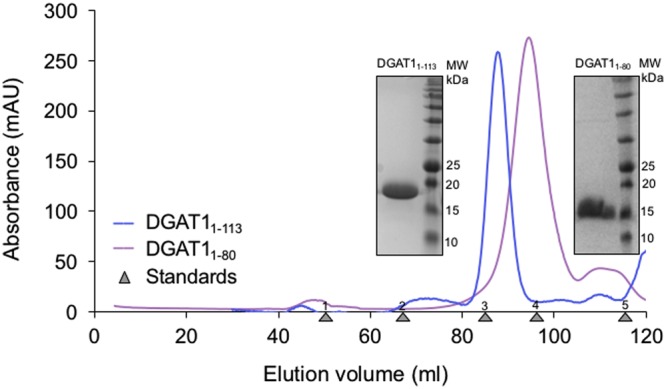
Size exclusion profiles of His-tagged constructs for the DGAT1 N-terminal domain (BnaDGAT1_1-113_) and the intrinsically disordered inhibition module containing region (BnaDGAT1_1-80_). Blue trace denotes BnaDGAT1_1-113_ and purple trace denotes BnaDGAT1_1-80_. Coomassie stained SDS-PAGE of eluted protein peaks are shown in the insets. Elution peak position for gel filtration standards are shown (1. Thyroglobulin, 670 kDa, 2. Aldolase, 158 kDa, 3. Ovalbumin, 44 kDa, 4. Myoglobulin, 17 kDa, and 5. VitB_12_, 1.3 kDa).

The full-length cytoplasmic domain, BnaDGAT1_1-113_, and the autoinhibitory domain, BnaDGAT1_1-80_, with N-terminal His-tags (Supplemental information), were recombinantly expressed in BL21(DE3) strain of *Escherichia coli* and purified using Ni-NTA affinity chromatography. The mobility of these recombinant proteins on SDS-PAGE was atypical as they migrated slower than expected, based on their expected molecular masses (16 kDa and 9 kDa, respectively) (Fig. 2 inset). This lower mobility, specifically observed with the recombinant N-terminal domains of BnaDGAT1_1-80_, is often observed in IDPs (eg. Juxtanodin) and attributed to the lack of hydrophobic amino acid residues and anomalous interaction with SDS^37,38^. It was observed that the purified proteins were prone to oligomerization and aggregation in a concentration and time dependent manner. Additionally, these purified constructs were susceptible to proteolysis despite the addition of protease inhibitors. In preliminary experiments, TEV protease was added to cleave His tag from BnaDGAT1_1-113_, but this required long incubation times (2 hours to overnight), after which degradation and nonspecific oligomers were observed when proteins were applied on a size exclusion column. Furthermore, concentrating the protein (>200 μM) after Ni-NTA affinity chromatography also led to oligomerization. To mitigate these issues, several steps were taken. Because milligram amounts of protein were required for the biophysical studies presented in this manuscript, the TEV protease cleavage step was omitted, and we worked with His-tagged DGAT1 proteins. Notably, the CD spectrum indicate the disordered nature of our untagged^23^ and His-tagged proteins in solution, however, as expected, differences in the spectral properties can be seen (Supplementary Figure 2). Due to its higher stability, the His-tagged protein was used for further biophysical studies since the tag is located at the disordered N-terminal region, which is distal to the folded C-terminal region and the primary ligand binding site^23^. Hence tag-induced secondary structure elements, if any, might not influence the ligand binding properties. Lastly, prepared protein samples were used immediately after concentration following size exclusion chromatography (SEC) to avoid aggregation upon storage at 4 °C.

Freshly purified samples of BnaDGAT1_1-113_ and BnaDGAT1_1-80_ yielded a monodisperse peak on SEC (Fig. 2). The hydrodynamic radii (Stoke’s radius, R_s_) for DGAT1 constructs were calculated from the standard curve using folded globular protein standards. The values were 2.2 nm and 1.7 nm for BnaDGAT1_1-113_ and BnaDGAT1_1-80_ respectively. The apparent molecular mass calculated from above was 21 kDa and 10.5 kDa respectively. Given the predicted non-globular (extended) nature of these proteins, the exact molecular mass and oligomeric state was calculated using in-line classical light scattering in tandem with SEC (SEC-MALLS) (Supplementary Figure 3, 4). These studies confirmed the molecular masses 13.9 kDa (BnaDGAT1_1-113_) and 8.6 kDa (BnaDGAT1_1-80_). The apparent molecular mass calculated from SEC-MALLS were lower than that calculated using SEC standards demonstrating the nonglobular and monomeric nature of the purified constructs. In addition, BnaDGAT1_1-113_ or BnaDGAT1_1-80_ preincubated with CoA or oleoyl-CoA were assessed using the above biophysical method to validate whether any change in conformation occurs in the presence of ligands. Peaks eluted at similar positions compared to their unbound forms suggested that the interaction with ligand did not cause a major change in the overall monomeric state or its hydrodynamic radius.

### BnaDGAT1_1-113_ and BnaDGAT1_1-80_ interacts with oleoyl-CoA or CoA with micromolar affinity

NMR titration experiments and docking studies have indicated that residues Arg96, Arg97, Arg99 and Glu100 of BnaDGAT1_81-113_ are involved in the interaction with CoA^23^. However, the thermodynamics of interaction of CoA ligands with the cytosolic domain of BnaDGAT1, or any other DGAT1, has not been reported. In preparation for isothermal titration calorimetry (ITC), standard buffer optimization was assessed using fluorescence-based thermal shift assay^39^, which demonstrated that BnaDGAT1_1-113_ was stable in sodium phosphate buffer (pH 7.5), with a T_m_ of ~38 °C (Supplementary Figure 5). Incubation with oleoyl-CoA at 1:1 molar ratio of ligand to protein resulted in a 10 degree increase in T_m_, indicating that this acyl-CoA binds to and stabilizes BnaDGAT1_1-113_. The protein both in apo and complex forms showed downward sloping characteristic aggregation profile in all other buffer conditions screened in the thermal shift assay.

**Figure 3:**
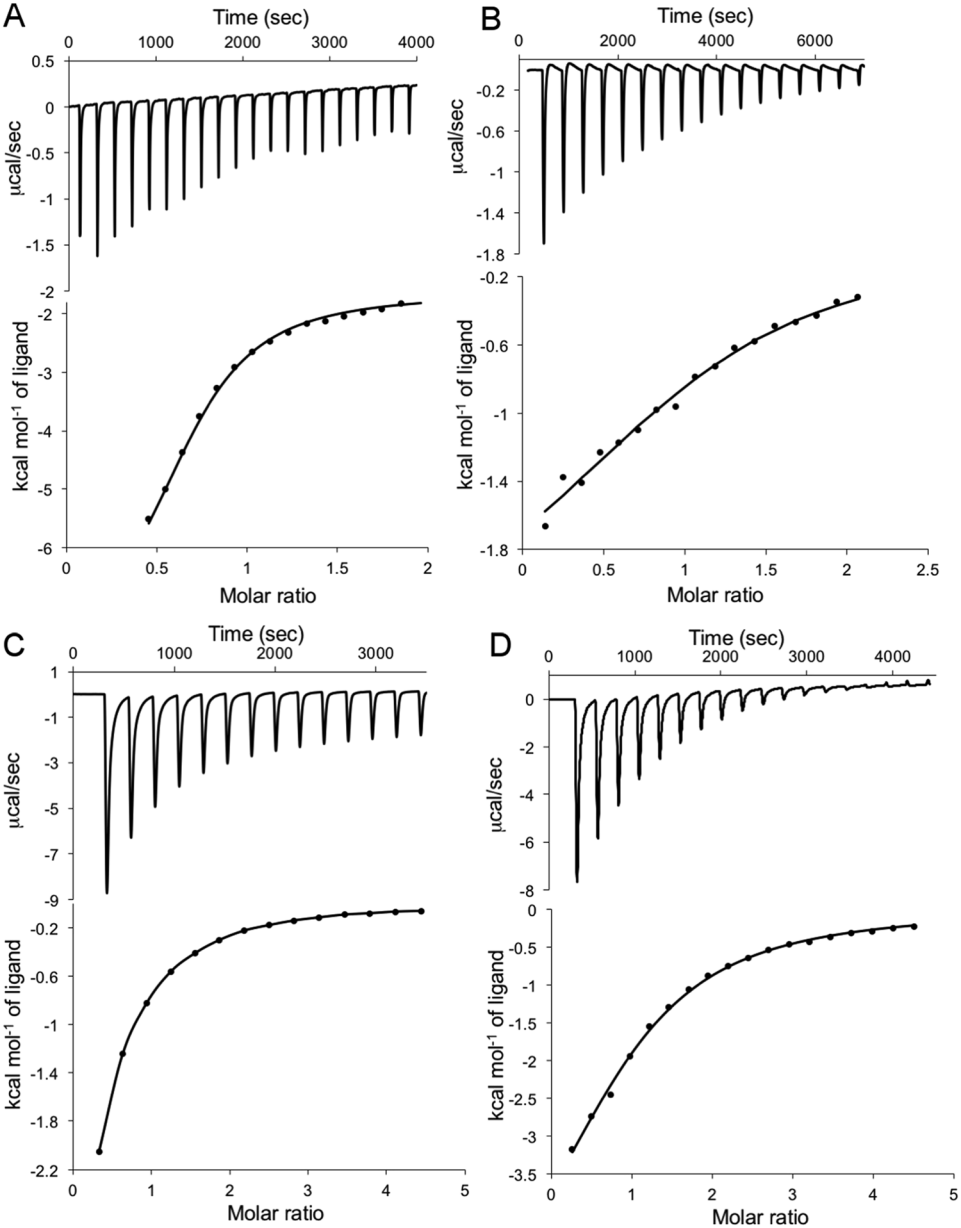
Isothermal titration calorimetric isotherms for interaction of N-terminal domain, BnaDGAT1_1-113_, and the inhibitory module containing region, BnaDGAT1_1-80_, with ligands. (**A**) Interaction of BnaDGAT1_1-113_ with oleoyl-CoA; (**B**) Interaction of BnaDGAT1_1-113_ with CoA; (**C**) Interaction of BnaDGAT1_1-80_ with oleoyl-CoA; (**D**) Interaction of BnaDGAT1_1-80_ with CoA. The top panels represent the baseline corrected raw data and the bottom panels show the integrated curve fit for titration of protein into ligand (oleoyl-CoA or CoA) to one set of sites.

ITC was performed to investigate the interaction thermodynamics of BnaDGAT1 N-terminal domain with acyl-CoA and CoA. Four independent systems were analyzed: BnaDGAT1_1-113_ or BnaDGAT1_1-80_ in combination with oleoyl-CoA or CoA. Our analysis revealed the interactions were exothermic and exhibited a stoichiometry of 1:1. Given the 1:1 stoichiometry for each protein truncation with the ligands tested, it is therefore likely that residues 1-80 coordinates with 81–113 to bind either oleoyl-CoA or CoA. The affinity of interaction of BnaDGAT1_1-113_ for oleoyl-CoA and CoA were 10 μM and 59 μM, respectively (Fig. 3A, B), indicating that the N-terminal domain of BnaDGAT1 has a higher affinity for thioester than free CoA. Interestingly, BnaDGAT1_1-80_ also interacted with both ligands but with lower affinity (K_d_ of 117 μM for oleoyl-CoA and K_d_ of 178 μM for CoA) (Fig. 3C, D). The thermodynamic parameters obtained for the above interactions were analyzed (Table 1). The interaction of BnaDGAT1_1-113_ with oleoyl-CoA is clearly an enthalpy-driven process likely due to water loss, indicating that conformational changes in one or both of the partners may occur. The other three systems under study demonstrated entropy-driven events, which could be due to a combination of the release of water molecules upon interaction with the ligand and a classical hydrophobic effect.

**Table 1:** Thermodynamic parameters obtained from isothermal titration calorimetry for BnaDGAT1 truncations.

Sample	N	Kd (µM)	∆H (kcal/mol)	T∆S (kcal/mol)	∆G (kcal/mol)
DGAT1_1-113_ + Oleoyl-CoA	1	10.00 ± 0.01	−8.0 ± 0.3	−0.34	−7.69
DGAT1_1-113_ + CoA	0.98 ± 0.08	60 ± 10	−2.8 ± 0.3	2.89	−5.66
DGAT1_1-80_ + Oleoyl-CoA	0.94 ± 0.02	120 ± 30	−2.2 ± 0.2	3.04	−5.24
DGAT1_1-80_ + CoA	1.10 ± 0.04	180 ± 10	−5.3 ± 0.3	−0.99	−6.28

N is the stoichiometry, **∆**G is calculated change in Gibb’s free energy, **∆**H is change in enthalpy, **∆**S is the change in entropy and K_d_ is the binding affinity.

### Circular dichroism reveals that ligand-binding results in gain of secondary structure in BnaDGAT1_1-113_ and BnaDGAT1_1-80_

CD was used to probe possible gains in secondary structure and folding in BnaDGAT1_1-113_ and BnaDGAT1_1-80_ as a consequence of binding oleoyl-CoA or CoA. The CD spectra of His-tagged BnaDGAT1_1-113_ is non-zero between 250-270 nm (Supplementary Figure 2) and gradually reaches zero at 274 nm. This anomaly is reproducible with different batches of purified protein and different CD spectrophotometers. Further the purified protein in CD buffer showed no aggregation on SEC, hence the above anomaly could not be explained. The secondary structural changes upon ligand binding will be observed between 190 nm – 222 nm, which is not a part of the above anomaly. Hence further CD studies to decipher secondary structural changes upon ligand binding was carried out.

**Figure 4:**
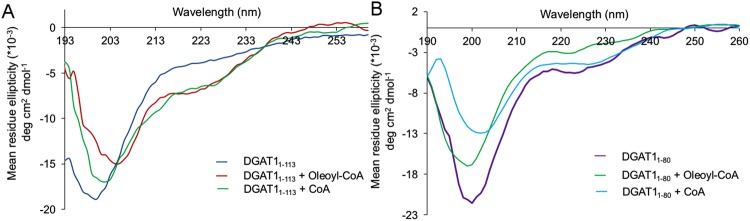
Circular dichroism (CD) profiles of various BnaDGAT1 constructs with and without ligands. (**A**) CD traces of BnaDGAT1_1-113_  in the presence and absence of oleoyl-CoA and CoA are shown; (**B**) CD traces of BnaDGAT1_1-80_  in the presence and absence of oleoyl-CoA and CoA are shown.

In the absence of ligand, both proteins exhibited a negative minimum close to 200 nm, a typical observation for disordered proteins^37^ (Fig. 4A,B). For ligand bound forms of BnaDGAT1_1-113_, a shift in the negative peak towards higher wavelengths was observed with the negative shoulder at 222 nm being more pronounced. This result indicates the existence of a minor α-helical region in BnaDGAT1_1-113_, which increases upon binding of oleoyl-CoA or CoA (Fig. 4A, Supplementary Table 1). The minor α-helical region could be the helix (L^103^-S^112^) observed in the NMR studies^23^. Contrary to the above, BnaDGAT1_1-80_ showed a decreased helicity upon ligand binding, along with a decrease in random coil nature as demonstrated by the decrease in negative ellipticity at ~200 nm (Fig. 4B). Overall, these observations suggest that there is some gain in helical structure for the entire segment (1-113) and reduction in the random coil nature of the shorter segment upon ligand binding.

### BnaDGAT1_1-113_ and BnaDGAT1_1-80_ exhibit extended structures with flexible properties

Synchrotron small-angle X-ray scattering (SAXS) experiments were performed to obtain detailed 3D structural information on this disordered region of DGAT1. SAXS data was collected after the protein had been passed over an inline SEC in order to separate the components of the mixture (Supplementary Figure 7). BnaDGAT1_1-113_ and its ligand-bound forms were monodisperse (Table 2). However, BnaDGAT1_1-80_ and its ligand bound states were problematic for SAXS data analysis; although they eluted at the position that corresponded to a monomeric form, aggregation was observed during the SAXS analysis suggesting that the samples may be prone to self-association and consequently aggregation right after the elution from the SEC.

**Table 2:** Small-angle x-ray scattering data collection and analysis for BnaDGAT1 truncations.

*Sample*	DGAT1_1-113_	DGAT1_1-113_ + Oleoyl-CoA	DGAT1_1-113_ + CoA
***Data collection***			
Beamline	SSRL BL4-2	SSRL BL4-2	SSRL BL4-2
Beam current	500 mA (5 min top-off)	500 mA (5 min top-off)	500 mA (5 min top-off)
Type of monochromator	Si(111)	Si(111)	Si(111)
Wavelength	1.127 Å (11 keV)	1.127 Å (11 keV)	1.127 Å (11 keV)
Beam defining slits size	0.3 mm (H) x 0.3 mm (v)	0.3 mm (H) x 0.3 mm (v)	0.3 mm (H) x 0.3 mm (v)
Sample-Detector distance	1.1 m	1.1 m	1.1 m
Detector	Rayonix MX225-HE	PILATUS3	PILATUS3
Pixel size	292 μm	172 μm	172 μm
q range*	0.011 − 0.67 Å^−1^	0.011 − 0.63 Å^−1^	0.011 - 0.63 Å^−1^
Sample cell size (quartz capillary)	1.5 mm in diameter	1.5 mm in diameter	1.5 mm in diameter
Type of experiment	SEC-SAXS	SEC-SAXS	SEC-SAXS
SEC column	Superdex 200 PC3.2/300	Superdex 200 Increase PC3.2/300	Superdex 200 Increase PC3.2/300
Chromatography system	GE	Thermo Fisher Scientific	Thermo Fisher Scientific
	Akta Ettan FPLC	UltiMate 3000 UHPLC	UltiMate 3000 UHPLC
Sample injection volume	50 µL	50 µL	50 µL
Sample concentration	15.0 mg/ml	20 mg/ml	20 mg/ml
Flow rate	0.05 ml/min	0.05 ml/min	0.05 ml/min
Temperature	293 K	293 K	293 K
Exposure time per frame	1 sec	1 sec	1 sec
Frames per experiment	600	500	500
**Guinier analysis**			
I(0)	2844.68 + /− 9.36	19.69 + /− 0.11	23.12 + /− 0.12
R_g_	32.14 + /− 0.82	34.03 + /− 1.63	33.41 + /− 1.34
**Software employed**			
*Primary data reduction*	SasTool	SasTool	SasTool
Data processing	PRIMUS	PRIMUS	PRIMUS
Guinier analysis	AUTORG/PRIMUS	AUTORG/PRIMUS	AUTORG/PRIMUS

^*^q = 4πsin(θ)/λ, where 2θ is the scattering angle.

**Figure 5:**
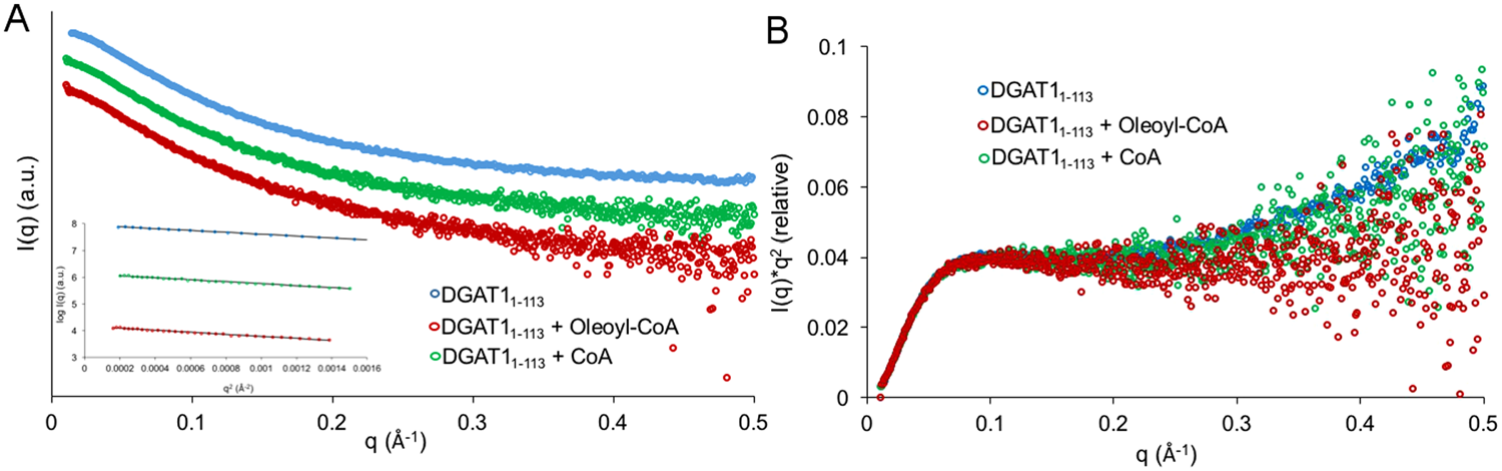
Size exclusion chromatography-Small angle X-ray scattering (SEC-SAXS) analysis for BnaDGAT1_1-113_, BnaDGAT1_1-113_ incubated with oleoyl-CoA and BnaDGAT1_1-113_ incubated with CoA. (**A**) The SAXS profiles are overlaid, accompanying Guinier plots (as inset). (**B**) Kratky plots for all of the above samples are overlaid visually depicting level of flexibility.

The scattering profiles, for the various forms of BnaDGAT1_1-113_, exhibited a typical profile of IDRs (Fig. 5A). The consecutive Guinier analyses over the elution and the elongated linearity on the Guinier plots indicated that the samples were well-separated from any aggregates and had no concentration-dependent inter-particle interactions (Fig. 5A inset and Supplementary Figure 7). The radius of gyration (R_g)_ derived from the Guinier analyses for apo- BnaDGAT1_1-113_ was 32.14 +/− 0.82 Å for the protein. Subtle differences for BnaDGAT1_1-113_ with different ligands were observed; indicating absence of overall large scale structural changes with this domain when incubated with acyl-CoA or CoA in solution. Given the experimental radius of gyration collected from Guinier analysis, we calculated the theoretical R_g_ using simple power-law relationship for intrinsically disordered proteins^40^ as stated below:

$${R}_{g}=2.49\times {N}^{0.509}$$

The value obtained from the above was 3.1 nm, which was close to our experimental value of 3.2 nm. The scattering intensity [I(q).q^2^] as a function of vector [q] denotes the Kratky plot, which is a qualitative analysis of the scattering profile^41,42^. The Kratky plots do not have maxima, but instead have a short plateau followed by a monotonic increase at higher value of [q]. This indicates the presence of a highly extended particle for BnaDGAT1_1-113_, thus confirming the intrinsically disordered nature of the N-terminal region of BnaDGAT1_1-113_ (Fig. 5B).

**Figure 6:**
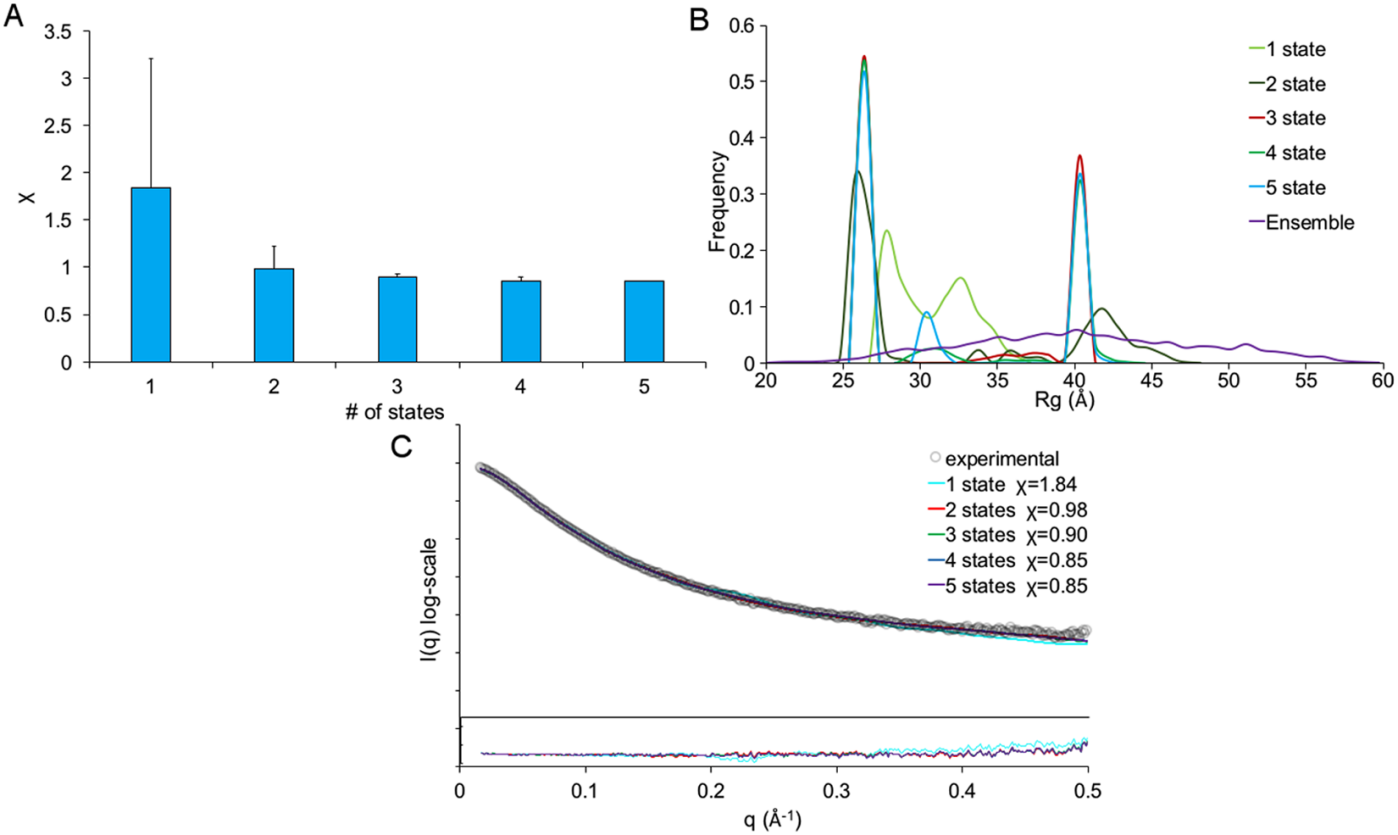
Small angle X-ray scattering modelling of BnaDGAT1_1-113_ using the program MultiFoXS. (**A**) The lowest χ scores for each of the N-state models (N = 1–5) are shown with error bars indicating the range of χ values. (**B**) Rg distributions for multi-state models of each size. (**C**) Comparison of SAXS profiles (experimental and computed) for the N-state models. The residual is shown in the inset.

**Figure 7:**
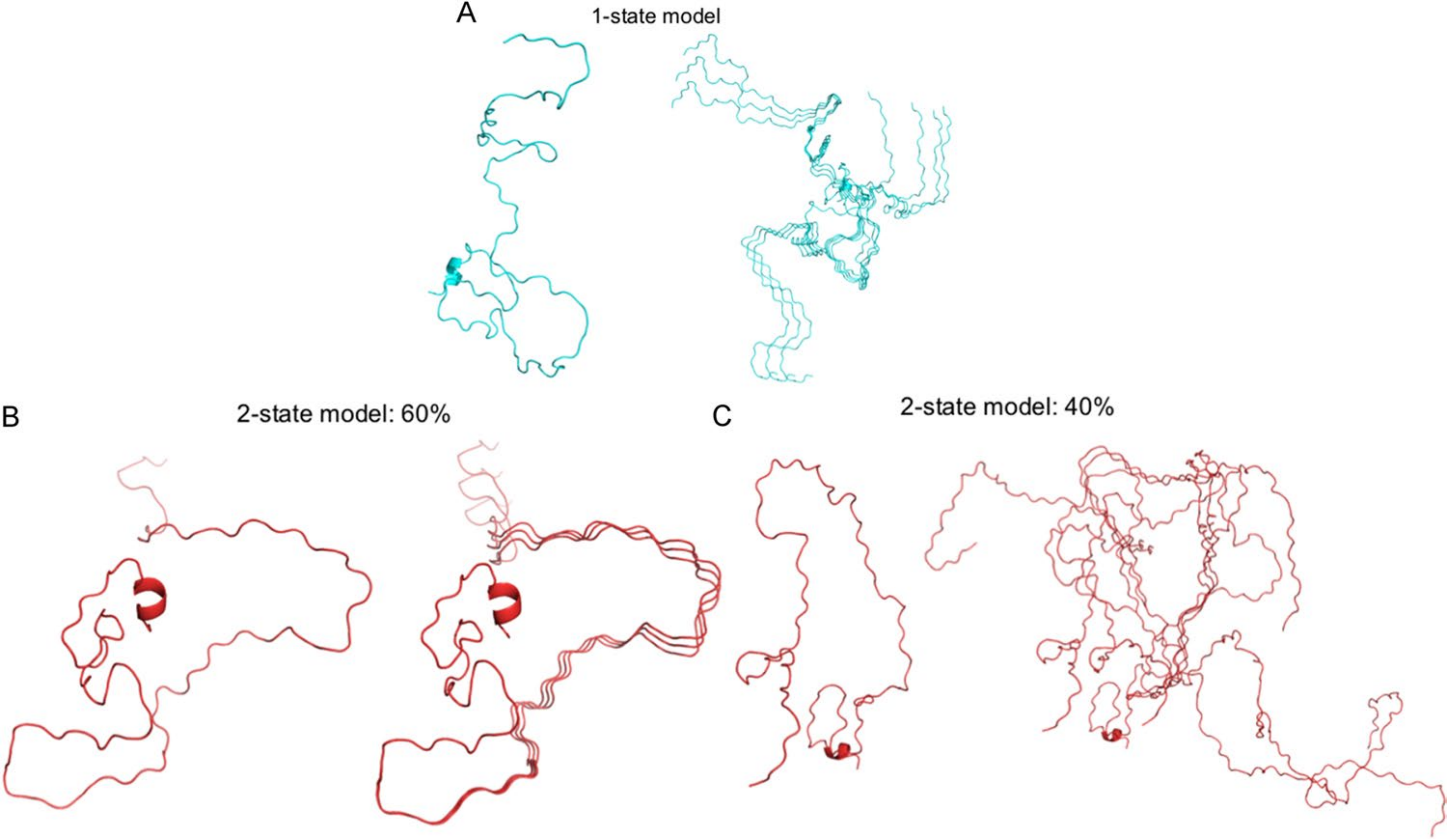
Conformations of the N-state models for BnaDGAT1_1-113_ from SAXS analysis. Both 1-state (**A**) and two-state models (**B** and **C**) are shown. Other models are shown in Supplementary figures. In each case, the best scoring model is shown on the left and the top 10 combinations (some of them are duplicated) are aligned on the right. The ratio of population is also indicated. See 3- to 5-state models in Supplementary Figures 8–10.

To reveal the SAXS model of the N-terminal domain of BnaDGAT1, our previous NMR structure^23^ was employed to generate a rigid model of residues 81–113, and then the program AllosMod-FoXS, which can fill missing fragments, was used to generate a full-length initial model for MultiFoXS. It is unlikely that the system does exist in one conformation as suggested by the Kratky plot (Fig. 5B); therefore, multi-state modeling with the SAXS profiles was performed using MultiFoXS. Rapid exploring Random Tree (RRT) method generated 10,000 conformations and their SAXS profiles were calculated. Further N-state model was enumerated with 1000 (for N = 1–2), 755 (for N = 3), 183 (for N = 4), and 25 (for N = 5) top scoring models for each N (Fig. 6A,B). The fits of the models against raw SAXS data were all in agreement, clearly demonstrating that increasing the number of the states provided better agreements from χ score = 1.84 to 0.85 (Fig. 6C). The results revealed the N-terminal domain BnaDGAT1_1-113_ exists in multiple extended conformations, with the top 10 scoring models at each state shown in (Fig. 7 and Supplementary Figure 8–10).

## Discussion

We have recently shown that the cytosolic N-terminal domain regulates the activity of the integral membrane-bound BnaDGAT1, and that acyl-CoA and CoA are positive and negative effectors of enzymatic activity, respectively^23^. Here, we conducted a biophysical study of the IDR of the soluble N-terminal domain of BnaDGAT1 and revealed its ability to gain secondary structure in the presence of allosteric effectors. Our sequence-based prediction analysis indicated that the N-terminal cytosolic domain of BnaDGAT1 (i.e., BnaDGAT1_1-113_) is intrinsically disordered. Our NMR structure of BnaDGAT1_81-113_ demonstrated that this is the minimal structural unit in the cytosolic domain consisting of a defined loop (Ala94-Pro102) followed by a short helix (Leu103-Ser112)^23^. Furthermore, bioinformatic analysis of the N-terminal domain indicates the presence of 14 potential phosphorylation sites (using Disorder enhanced phosphorylation predictor; Supplementary Figure 1C) and a myristoylation site. The presence of a myristoylation site in DGAT1 has not been discussed previously, yet it is interesting because it opens up the possibility of the N-terminal domain of BnaDGAT1 being anchored to the phospholipid bilayer, thus controlling motion of the domain in the cytosol as a mode of regulation. Similarly, a myristate group is known to adhere the C-terminal region of the SH4 domain of src-kinase to the lipid bilayer^43^. The lack of structure, the potential for posttranslational modification, and the important role this domain plays in regulation of the activity of the membrane-embedded domain of BnaDGAT1, led us to investigate its interaction with effectors in more detail.

The major challenge posed in this study was the high propensity of aggregation and degradation of this IDR. Thermal shift assay was used to circumvent the obstacle by identifying the optimal buffer conditions wherein the proteins could remain stable during analysis, and thus allow for the binding studies and structural analyses to be conducted. Furthermore, fresh protein preparation was crucial for the study.

In our previous study, we demonstrated with NMR titration analysis, docking studies and mutagenesis that BnaDGAT1_81-113_ is capable of interacting with CoA and acyl-CoA^23^. In the current study, we explored the thermodynamics of interaction of BnaDGAT1_1-113_ and BnaDGAT1_1-80_ with oleoyl-CoA and CoA using ITC. Although a previous study has shown that BnaDGAT1_1-116_His_6_ (based on isoform BnaA.DGAT1.b), with hexa-His tag at the carboxyl end, leads to a protein segment that self-associates^28^, the N-terminally tagged recombinant protein segments used in this study favored the monomer in solution. During SEC used to purify these recombinant proteins, it was noted that there was, however, a tendency for these to aggregate if the sample concentration applied to the SEC column was above 200 μM. Our analyses show that the N-terminal domain of BnaDGAT1 has higher binding affinity for oleoyl-CoA than CoA. This is in line with our previously conducted kinetic analysis on the above region^23^. Interestingly, BnaDGAT_1-80_ is also capable of interacting with CoA and oleoyl-CoA, but there is a ten-fold decrease in affinity compared to the full cytosolic domain BnaDGAT1_1-113_. Our previous *in vivo* analysis of truncated forms of BnaDGAT1 suggested that BnaDGAT1_1-80_ represents an autoinhibitory region of the cytosolic domain of BnaDGAT1^23^. The fact that this region also interacts with CoA or acyl-CoA supports the above proposed regulatory role of this region.

Early studies on the N-terminal domain of BnaDGAT1 suggested that they primarily formed monomers, with dimer and tetramer species observed with crosslinking. SEC analysis of the N-terminal domain also supporting self-association, however at this time the nature of the intrinsic disorder in this region was not known^28^. More recently, we further demonstrated full-length BnaDGAT1 forms a dimer in yeast membranes^23^. Upon truncation of this disordered region, the first 80 residues, of the cytoplasmic N-terminus of BnaDGAT1, crosslinking was no longer observed, suggesting dimerization occurs via this N-terminal region^23^. Furthermore, the N-terminal domain of murine DGAT1 was also shown to be important for dimerization and tetramerization^28^. It should be noted, however, that there are slight differences in sequence between the N-terminal domains of these two species (25% sequence identity). This agrees with previous findings that long IDRs have high sequence variability^44^. Nonetheless, we previously found that the nature of the amino acid residues within the N-terminal domain identified to bind CoA is conserved between animal and plant sequences^23^. In the current study, however, where we study the first eighty residues, we primarily observe a monomeric species for the N-terminal region of BnaDGAT1. Taken together, we suggest that under the current conditions the BnaDGAT1_1-80_ region alone is not sufficient for dimerization. It is possible that tethering to the membrane allows for close proximity of the cytoplasmic N-terminal residues, which may facilitate dimerization. It is therefore likely that the N-terminal domain structure is influenced by the presence of the DGAT1 membrane domain or the lipid bilayer interface. Indeed, we have recently demonstrated that the negatively charged lipid phosphatidate in mixed micelles enhanced BnaDGAT1 activity, and this activation was attributed to the interaction of the N-terminal domain of BnaDGAT1 with this lipid^45^.

The BnaDGAT1 truncations used in the current study involved N-terminal tagging of the first 80 amino acid residues of isoform BnaC.DGAT1.a, whereas an earlier study was based on a C-terminally tagged BnaA.DGAT1.1b_1-116_^28^. In addition, although the amino acid sequences of BnaC.DGAT1.a and BnaA.DGAT1.b exhibit a very high level of identity, most differences in sequence occur in the protein segment encoded by the first exon^46^. Thus, the nature of truncation tagging (N-terminal versus C-terminal) and/or differences in sequence may have influenced possible oligomerization versus preservation of the monomeric species. We conclude, given our current understanding of this region from this work and our more recent papers, that the N-terminus is the preferred location to insert an affinity tag to obtain a stable homogeneous species for biophysical characterization and given the structural features at the C-terminus of the N-terminal domain. Interestingly, adding an N-terminal tag to amino acid sequences representing native BnaDGAT1 isoforms has previously been shown to mask the deleterious influence of the native N-terminal sequences, resulting in increased production of recombinant BnaDGAT1 polypeptides *in vivo* in a yeast system^42^. The resulting yeast microsomes also exhibited an increase in *in vitro* DGAT activity of to about 150-fold.

Owing to their inherent flexibility, very few biophysical techniques can be implemented to explore the structural details of IDRs. The increase in helix propensity of BnaDGAT1_1-113_ upon titration with trifluoroethanol had been previously reported^23^. Trifluoroethanol is known to stabilize protein secondary structure by lowering the dielectric constant of the solution thus mimicking a ligand bound state^47^. Our CD results indicate that indeed there is a gain in helicity upon ligand binding in BnaDGAT1_1-113_ and a decrease in random coil structure in case of BnaDGAT1_1-80_. It is interesting to note that the CD profile of full length BnaDGAT1^23^ exhibited predominantly helical nature and the truncated N-terminal fragments (BnaDGAT1_1-113_ and BnaDGAT1_1-80_) used in the previous^23^ as well as current study indicate predominantly disordered structure. This could again indicate that the presence of membrane domain and/or lipid bilayer could promote the structure of this cytosolic domain.

For 3D-structural analysis, SAXS data collection was carefully performed in order to minimize radiation damage and concentration-dependent inter-particle interactions, followed by further assessment of background-subtracted scattering profiles. Because of the intrinsically disordered nature of the hydrophilic N-terminal domain of BnaDGAT1, conformational heterogeneity made the data interpretation challenging. Our multi-state ensemble modeling using the program MultiFoXS fitted well to the experimental profile. This is consistent with the SEC profile that the samples are all monomeric in solution. The resulting over-fitting was avoided by computing ensembles of multi-state models that fitted the SAXS data, thus highlighting the conserved feature, which was the C-terminal region of BnaDGAT1_1-113_. Transient local secondary structure could be induced upon interaction with effectors while preserving the extended conformation of the protein in solution. However, these minor changes are not typically visualized using SAXS with IDRs containing extended conformation and high structural heterogeneity. This scenario has been previously observed with the adaptor protein, Juxtanodin^38^.

It has been previously reported that IDRs can stochastically fluctuate among multiple states such as coil-like or one with localized secondary structures^48^. The latter have been reported as molecular recognition elements (MoRFs), known to play a role in binding events^49,50^. MoRFs could fold as helices observed in short amphipathic stretches within long disordered sequences, β-strand^51^, or form irregular structures. A correlation between the SEC-MALLS and SEC-SAXS data is observed as the hydrodynamic radius and the radius of gyration did not change substantially upon incubation with the ligands. Our CD experiments indicate, however, that the binding event is subtly coupled with folding. Although the overall tertiary structure is in the extended conformation, there is a slight gain in the local secondary structure upon incubation with ligands. The interaction with the ligands could lead to formation of transient secondary structure. Such intricate secondary structural changes, however, are not large enough to be captured at present with low resolution SAXS data.

The IDR BnaDGAT1_1-80_ is unstructured according to CD experiments and is predicted to have a cluster of phosphorylation sites, reflecting a possible role in the regulation of the enzyme by the N-terminal domain. As an effect of the structural heterogeneity, the IDR in BnaDGAT1 could form modular assemblies of independently foldable regions, partially folded regions, or regions that never fold. Such a scenario has been observed in nuclear pore complexes where the IDRs containing multiple Phe-Gly motifs adopt various conformations for specific functions^48,52^.

The structural adaptability of IDRs facilitates their interaction with diverse partners with overlapping binding sites, a consequence of its linear conformation. This could provide an increased interaction surface area and avoid steric clashes. Consequentially, the speed and efficiency of the above interactions are also increased because of less stringent spatial orientation requirements. It has also been observed that IDRs could adopt different local secondary structures upon binding to different binding partners. The disordered C-terminal domain of p53 forms helix, sheet, or two different types of coils upon interaction with four different targets^53^. Additionally, IDRs are known to interact with their targets with high specificity and low affinity, which is also observed in our case as the binding occurs with effectors in micromolar range. This rapid association, initiation of signaling and easy dissociation allows the IDRs to coordinate regulatory events in space and time. As mentioned previously, the level of BnaDGAT1 activity during seed development can have a substantial effect on the flow of carbon into TAG. Considering the presence of multiple phosphorylation sites necessary for signaling^45^, and the ability to interact with acyl-CoA and/or CoA, this work adds to the growing body of literature that the cytoplasmic N-terminal domain of BnaDGAT1 not only regulates the enzyme activity by interacting with multiple effector proteins and ligands but also could aid in metabolic “pathway crosstalk” with nonrandom conformational preferences upon association with putative partners. Thus, this work sets the foundation for identifying other interactors of the IDR of BnaDGAT1 and other forms of the enzyme from various organisms that produce TAG.

## Methods

### In silico analysis

The analysis for the folding properties of cytoplasmic N-terminal domain of BnaDGAT1 was performed using DisoPred^31^, Globplot2^32^ and FoldIndex^33^. Disorder enhanced phosphorylation predictor^35^ was used to predict the potential phosphorylation sites in this domain.

### Preparation and purification of BnaDGAT1 truncations

The BL21(DE3) strain of *E*. *coli* (Invitrogen) was used as an expression system for recombinant pET16b encoding 10 x His-BnaDGAT1_1-113_ or 6 x His-BnaDGAT1_1-80_. Genetic constructs were designed such that each recombinant polypeptide segment contained an N-terminal tag as shown in the supplemental data. Preinnoculum was prepared by inoculation of single colony to 100 ml of Luria Broth media with 100 mg/mL ampicillin grown for overnight in a rotary shaker. Overnight culture (20 mL) was used to inoculate 1 litre of LB with ampicillin and 6 litre of inoculated media was grown at 37 °C until the optical density (OD_600_) reached 0.7 to 0.8. Expression of recombinant protein was induced by addition of 1 mM of isopropyl-b-thiogalactopyranoside (IPTG). After 5 hrs of incubation at 30 °C, the cells were harvested by centrifugation at 4000 × *g* (10 min, 4 °C), resuspended in 100 ml of buffer A (50 mM Tris pH 8.0, 500 mM NaCl and 20% glycerol) supplemented with protease inhibitor tablet (Roche scientific). The lysate was emulsified using Avestin Emulsiflex and further clarified by centrifugation (10000 × *g*, 1 hr, 4 °C). Ni-NTA affinity chromatography was performed using Ni-NTA beads (Qiagen) incubated with the clarified lysate. The protein was eluted using a gradient mix of buffer A and buffer B (50 mM Tris pH 8.0, 500 mM NaCl, 20% (v/v) glycerol and 2 M imidazole). The presence of purified protein was observed on Coomassie stained SDS-PAGE. To further obtain monodispersed preparation, SEC was performed in a Superdex 200 (16/60) (GE Healthcare) with Superdex buffer containing 25 mM sodium phosphate pH 8.0, 300 mM NaCl, 5% (v/v) glycerol.

### Multi-angle laser light scattering (MALLS)

The central peak fractions after the size exclusion column were used for molecular mass determination by inline size-exclusion chromatography-multi-angle laser light scattering (SEC-MALLS). Samples, 50 μl, were loaded at 5 mg/ml onto the SEC-MALLS and the above superdex buffer was used for elution and for differential refractometry. Data was analyzed with ASTRA V software (Wyatt Technology). BSA was used to normalize the MALLS detector. Experiments were performed at 25 °C on an FPLC-managed Superdex 200 10/300 size exclusion chromatography column (GE Healthcare), coupled to a Wyatt DAWN EOS light scattering detector, Optilab^®^ rEX Refractive Index Detector, QELS (Quasi Elastic Light Scattering), UV detector (Wyatt Technology).

### Thermal shift assay

In order to characterize the stability of the BnaDGAT1 N-terminal domain in different aqueous conditions, thermofluor assay using SYPRO Orange dye was conducted^54^. Screens were designed with a varied range of buffers pH (3.0 - 10.0) combined with different concentrations of sodium chloride (0 mM, 100 mM, 300 mM, 500 mM), with and without 5% (v/v) glycerol. Aliquots, 2 µl, of protein (5 mg/ml) were mixed with equal volume of 1X dye solution. Aliquots, 2 µl, of the above mixture was added to 18 µl of each screen solution placed in 96 well plates. Each reaction was performed in triplicates. Fluorescence was monitored on an Applied Biosystems 7500 FAST RealTime PCR System with an excitation range of 510–530 nm while the temperature was held for 1 min per degree from 24–95 °C. The emission signal at 567–596 nm was used for analysis. Similar analysis was performed with protein incubated with oleoyl-CoA at different molar ratios (1:1, 1:2, 1:5 and 1:10). The shift in melting temperature (T_m_) indicated binding.

### Isothermal titration calorimetry

Binding studies for both constructs were performed using VP-ITC Microcal Instruments (GE Healthcare). Oleoyl-CoA or CoA was dissolved in ITC buffer (25 mM phosphate pH 7.5, 300 mM NaCl and 5% glycerol) at concentration of 130 µM and the resulting solution was placed in the analysis cell. Protein at 1.13 mM was titrated against the cell. Titrations were carried out at 20 °C using 10 µL of syringe solution injected at an interval of 200 s. Control experiments were performed by injecting protein into the cell containing the buffer to rule out the possibility of self association of protein at high concentration (Supplementary Figure 6). The data fitting was performed using non-linear least square curve-fitting algorithm (Microcal Origin). The best fit to the data to calculate the binding affinity *K*_*d*_ was obtained using *χ*^2^ minimization on a model assuming a single set of binding sites. Three floating variables: stoichiometry (N), binding constant (K_d_) and the change in enthalpy of interaction (∆H) were finally obtained^55^. For the system containing BnaDGAT1_1-113_ and oleoyl-CoA, MicroCal ITC200 (GE Healthcare) was used (2 µl injection, 200 seconds spacing and 20 °C) and the data obtained was analyzed using SEDPHAT^56^. The experiments were performed in duplicate. Data shown is mean with standard error.

### Circular dichroism

Jasco-810 spectropolarimeter was used to measure the far-UV CD spectra corresponding to peptide bond absorption at 10 °C. Spectra were collected for 10 µM of protein in 10 mM of sodium phosphate buffer pH 7.3 in a Quartz SUPRASIL cuvette (Hellma) with a path length of 1 mm. Measurements were made with an increment step of 0.5 nm, an integration time of 4 s per step and a bandwidth of 2 nm. Each spectrum was an average of 5 accumulations. The protein was incubated with ligand at 1:1 molar ratio. The signal due to buffer alone and buffer with ligand was subtracted from that of the protein and protein with ligand respectively. The proportions of secondary structures of the protein was estimated from the [Θ] values between 190 and 240 nm using the DichroWeb server (http://dichroweb.cryst.bbk.ac.uk/html/home.shtml)^57^ and the CDSSTR algorithm^58^ and SP175 reference data set.

### Solution X-ray scattering studies

SEC-SAXS studies were performed at SSRL Beamline 4–2 to mitigate aggregation, as described previously^59,60^. The details were summarized in Table 2. Briefly, Superdex 200 PC3.2/300 (GE Healthcare, Wisconsin USA) was used with a flowrate of 0.05 mL/min. The column was equilibrated with Superdex buffer. SAXS data were collected with nominal sample-to-detector distance of 1.1 m, at 11 keV, with an exposure time of 1 sec per image for every 5 secs. In order to keep sample cell clean, x-ray scattering images were only taken at the first ~100 images, for blank images, and around main peak of interest. SasTool (http://ssrl.slac.stanford.edu/~saxs/analysis/sastool.htm) was used for scaling, azimuthal integration, averaging of individual scattering images after inspection, and background subtraction; the intial 100 images were used for background scattering profile^56^. The script hplcplots, available at SSRL beamline 4–2, was then used for consecutive Guinier analysis, implemented in the program AUTORG^61^ for assessing data quality (e.g., radiation damage and cleanness of sample cell) and for averaging profiles every 5 frames. The final averaged curves were selected, following further assessment for inter-particle interactions and/or aggregations using the program PRIMUS^62^.

Since initial analysis indicated high flexibility of the BnaDGAT1_1-113_, the multi-state modeling was performed using the program MultiFoSX, which has been specially designed for samples showing conformational heterogeneity^63^. To generate an initial model for MultiFoXS, the previously reported NMR structure^23^ was employed for a rigid model of residues 81–113 and then the program AllosMod-FoXS (https://modbase.compbio.ucsf.edu/allosmod-foxs/) was used to fill other missing fragments. 10,000 conformations were generated for conformational sampling. The SAXS profile of each conformation is calculate for SAXS scoring and then multi-state model enumeration is performed iteratively. It simulated up to 5-state models and displayed top 100 and 25 best scoring models for 1–4-state and 5-state, respectively.

## Electronic supplementary material

Supplementary information

## References

[1] Wright PE, Dyson HJ (2015). Intrinsically disordered proteins in cellular signaling and regulation. Nature reviews. Molecular cell biology.

[2] Dunker AK, Brown CJ, Lawson JD, Iakoucheva LM, Obradovic Z (2002). Intrinsic disorder and protein function. Biochemistry.

[3] Pazos, F., Pietrosemoli, N., García-Martín, J. A. & Solano, R. Protein intrinsic disorder in plants. *Frontiers in Plant Science* **4**, 10.3389/fpls.2013.00363 (2013). 10.3389/fpls.2013.00363 PMC3770944 24062761

[4] Uversky VN (2013). A decade and a half of protein intrinsic disorder: Biology still waits for physics. Protein Science : A Publication of the Protein Society.

[5] Ward JJ, Sodhi JS, McGuffin LJ, Buxton BF, Jones DT (2004). Prediction and functional analysis of native disorder in proteins from the three kingdoms of life. Journal of molecular biology.

[6] Babu MM, van der Lee R, de Groot NS, Gsponer J (2011). Intrinsically disordered proteins: regulation and disease. Current opinion in structural biology.

[7] Uversky VN, Gillespie JR, Fink AL (2000). Why are “natively unfolded” proteins unstructured under physiologic conditions?. Proteins.

[8] Garner E, Cannon P, Romero P, Obradovic Z, Dunker AK (1998). Predicting Disordered Regions from Amino Acid Sequence: Common Themes Despite Differing Structural Characterization. *Genome informatics*. Workshop on Genome Informatics.

[9] Williams, R. M. *et al*. The protein non-folding problem: amino acid determinants of intrinsic order and disorder. *Pacific Symposium on Biocomputing*. *Pacific Symposium on Biocomputing*, 89–100 (2001). 10.1142/9789814447362_0010 11262981

[10] Romero P (2001). Sequence complexity of disordered protein. Proteins.

[11] Uversky VN, Dunker AK (2010). Understanding protein non-folding. Biochimica et biophysica acta.

[12] Malhis N, Jacobson M, Gsponer J (2016). MoRFchibi SYSTEM: software tools for the identification of MoRFs in protein sequences. Nucleic Acids Research.

[13] Uversky VN (2013). MultIDIMensionality of IDIMs: Intrinsic disorder in autoinhibition. Structure.

[14] Bah A, Forman-Kay JD (2016). Modulation of intrinsically disordered protein function by post-translational modifications. Journal of Biological Chemistry.

[15] Diella F (2008). Understanding eukaryotic linear motifs and their role in cell signaling and regulation. Frontiers in bioscience : a journal and virtual library.

[16] Fuxreiter M (2008). Malleable machines take shape in eukaryotic transcriptional regulation. Nature chemical biology.

[17] Galea CA, Wang Y, Sivakolundu SG, Kriwacki RW (2008). Regulation of cell division by intrinsically unstructured proteins: intrinsic flexibility, modularity, and signaling conduits. Biochemistry.

[18] Paul C, Rosenbusch JP (1985). Folding patterns of porin and bacteriorhodopsin. The EMBO journal.

[19] Rees DC, Eisenberg D (2000). Turning a reference inside-out: commentary on an article by Stevens and Arkin entitled: “Are membrane proteins ‘inside-out’ proteins”? (Proteins 1999; 36: 135–143). Proteins.

[20] Xue B, Li L, Meroueh SO, Uversky VN, Dunker AK (2009). Analysis of structured and intrinsically disordered regions of transmembrane proteins. Molecular bioSystems.

[21] Minezaki Y, Homma K, Nishikawa K (2007). Intrinsically disordered regions of human plasma membrane proteins preferentially occur in the cytoplasmic segment. Journal of molecular biology.

[22] Bürgi J, Xue B, Uversky VN, van der Goot FG (2016). Intrinsic disorder in transmembrane proteins: Roles in signaling and topology prediction. Plos One.

[23] Caldo, K. M. P. *et al*. Diacylglycerol acyltransferase 1 is regulated by its hydrophilic N-terminal domain in response to allosteric effectors. *Plant Physiology*, 10.1104/pp.17.00934 (2017). 10.1104/pp.17.00934 PMC5619907 28827454

[24] Weselake RJ (2008). Metabolic control analysis is helpful for informed genetic manipulation of oilseed rape (Brassica napus) to increase seed oil content. Journal of Experimental Botany.

[25] Caldo KMP, Greer MS, Chen G, Lemieux MJ, Weselake RJ (2015). Purification and properties of recombinant Brassica napus diacylglycerol acyltransferase 1. FEBS Letters.

[26] Weselake RJ (2009). Increasing the flow of carbon into seed oil. Biotechnology advances.

[27] McFie PJ, Stone SL, Banman SL, Stone SJ (2010). Topological orientation of acyl-CoA:diacylglycerol acyltransferase-1 (DGAT1) and identification of a putative active site histidine and the role of the N terminus in dimer/tetramer formation. Journal of Biological Chemistry.

[28] Weselake RJ (2006). Acyl-CoA-binding and self-associating properties of a recombinant 13.3 kDa N-terminal fragment of diacylglycerol acyltransferase-1 from oilseed rape. BMC Biochemistry.

[29] Siloto RMP (2008). An N-terminal fragment of mouse DGAT1 binds different acyl-CoAs with varying affinity. Biochemical and Biophysical Research Communications.

[30] Roesler K (2016). An improved variant of soybean type 1 diacylglycerol acyltransferase increases the oil content and decreases the soluble carbohydrate content of soybeans. Plant Physiology.

[31] Jones DT, Cozzetto D (2015). DISOPRED3: precise disordered region predictions with annotated protein-binding activity. Bioinformatics.

[32] Linding R, Russell RB, Neduva V, Gibson TJ (2003). GlobPlot: exploring protein sequences for globularity and disorder. Nucleic Acids Res.

[33] Prilusky J (2005). FoldIndex©: a simple tool to predict whether a given protein sequence is intrinsically unfolded. Bioinformatics.

[34] Nakagami H (2010). Large-scale comparative phosphoproteomics identifies conserved phosphorylation sites in plants. Plant Physiology.

[35] Xie Y (2016). GPS-Lipid: a robust tool for the prediction of multiple lipid modification sites. Scientific reports.

[36] Walsh CT, Garneau-Tsodikova S, Gatto GJ (2005). Protein posttranslational modifications: The chemistry of proteome diversifications. Angewandte Chemie International Edition.

[37] Tompa P (2002). Intrinsically unstructured proteins. Trends in Biochemical Sciences.

[38] Ruskamo S (2012). Juxtanodin is an intrinsically disordered F-actin-binding protein. Scientific reports.

[39] Huynh K, Partch CL (2015). Analysis of protein stability and ligand interactions by thermal shift assay. Current protocols in protein science.

[40] Marsh JA, Forman-Kay JD (2010). Sequence Determinants of Compaction in Intrinsically Disordered Proteins. Biophysical Journal.

[41] Bernado P, Svergun DI (2012). Structural analysis of intrinsically disordered proteins by small-angle X-ray scattering. Molecular bioSystems.

[42] Cordeiro TN (2017). Small-angle scattering studies of intrinsically disordered proteins and their complexes. Current opinion in structural biology.

[43] Perez Y (2013). Lipid binding by the Unique and SH3 domains of c-Src suggests a new regulatory mechanism. Scientific reports.

[44] Brown CJ (2002). Evolutionary rate heterogeneity in proteins with long disordered regions. Journal of molecular evolution.

[45] Caldo, K. M. P. *et al*. Diacylglycerol acyltransferase 1 is activated by phosphatidate and inhibited by SnRK1-catalyzed phosphorylation. *The Plant Journal**In Press* (2018). 10.1111/tpj.14029 30003607

[46] Greer MS (2015). Engineering increased triacylglycerol accumulation in Saccharomyces cerevisiae using a modified type 1 plant diacylglycerol acyltransferase. Applied microbiology and biotechnology.

[47] Gast K, Zirwer D, Muller-Frohne M, Damaschun G (1999). Trifluoroethanol-induced conformational transitions of proteins: insights gained from the differences between alpha-lactalbumin and ribonuclease A. Protein Science : A Publication of the Protein Society.

[48] van der Lee R (2014). Classification of Intrinsically Disordered Regions and Proteins. Chemical Reviews.

[49] Wright PE, Dyson HJ (2009). Linking folding and binding. Current opinion in structural biology.

[50] Mohan A (2006). Analysis of molecular recognition features (MoRFs). Journal of molecular biology.

[51] Remaut H, Waksman G (2006). Protein–protein interaction through β-strand addition. Trends in Biochemical Sciences.

[52] Yamada J (2010). A bimodal distribution of two distinct categories of intrinsically disordered structures with separate functions in FG nucleoporins. Molecular & cellular proteomics : MCP.

[53] Hayashi T, Oshima H, Yasuda S, Kinoshita M (2015). Mechanism of one-to-many molecular recognition accompanying target-dependent structure formation: For the tumor suppressor p53 protein as an example. The journal of physical chemistry. B.

[54] Grøftehauge MK, Hajizadeh NR, Swann MJ, Pohl E (2015). Protein–ligand interactions investigated by thermal shift assays (TSA) and dual polarization interferometry (DPI). Acta Crystallographica Section D: Biological Crystallography.

[55] Leavitt S, Freire E (2001). Direct measurement of protein binding energetics by isothermal titration calorimetry. Current opinion in structural biology.

[56] Zhao H, Piszczek G, Schuck P (2015). SEDPHAT – a platform for global ITC analysis and global multi-method analysis of molecular interactions. Methods (San Diego, Calif.).

[57] Whitmore L, Wallace BA (2008). Protein secondary structure analyses from circular dichroism spectroscopy: methods and reference databases. Biopolymers.

[58] Sreerama N, Woody RW (2000). Estimation of protein secondary structure from circular dichroism spectra: comparison of CONTIN, SELCON, and CDSSTR methods with an expanded reference set. Anal Biochem.

[59] Matsui T (2014). Structural basis of the pH-dependent assembly of a botulinum neurotoxin complex. Journal of molecular biology.

[60] Callaway DJE (2017). Controllable activation of nanoscale dynamics in a disordered protein alters binding kinetics. Journal of molecular biology.

[61] Petoukhov MV, Konarev PV, Kikhney AG, Svergun DI (2007). ATSAS 2.1 - towards automated and web-supported small-angle scattering data analysis. Journal of Applied Crystallography.

[62] Konarev PV, Volkov VV, Sokolova AV, Koch MHJ, Svergun DI (2003). PRIMUS: a Windows PC-based system for small-angle scattering data analysis. Journal of Applied Crystallography.

[63] Schneidman-Duhovny D, Hammel M, Tainer JA, Sali A (2016). FoXS, FoXSDock and MultiFoXS: Single-state and multi-state structural modeling of proteins and their complexes based on SAXS profiles. Nucleic Acids Research.

