# Supplementary material for: Intrinsic disorder in the regulatory N-terminal domain of diacylglycerol acyltransferase 1 from Brassica napus
Source: Sci Rep. 2018 Nov 12;8:16665. doi: 10.1038/s41598-018-34339-1 (PMC6232145; doi:10.1038/s41598-018-34339-1)
Supplement: Supplementary file 1 — Supplementary information [file 41598_2018_34339_MOESM1_ESM.docx]

**Intrinsic disorder in the regulatory N-terminal domain of diacylglycerol acyltransferase 1 from *Brassica napus***

Rashmi Panigrahi^1^, Tsutomu Matsui^2^, Andrew H. Song^1^, Kristian Mark P. Caldo^3^, Howard S. Young^1^, Randall J. Weselake^3^ and M. Joanne Lemieux^1*^

^1^Department of Biochemistry, University of Alberta, Edmonton, Alberta, Canada T6G 2H7;

^2^Stanford Synchrotron Radiation Lightsource, SLAC National Accelerator Laboratory, Stanford University, Menlo Park, CA 94025, USA;

^3^Department of Agricultural, Food and Nutritional Science, University of Alberta, Edmonton, Alberta, Canada T6G 2P5

*Corresponding author: [mlemieux@ualberta.ca](mailto:mlemieux@ualberta.ca)

**Supplementary information:**

The amino acid sequences for the two His-tagged constructs are as below:

The resulting amino acid sequence for BnaDGAT_1-113_ is:  MGHHHHHHHHHHSSGHIEGRHMENLYFQGMEILDSGGVTMPTENGGADLDTLRHRKPRSDSSNGLLPDSVTVSDADVRDRVDSAVEDTQGKANLAGENEIRESGGEAGGNVDVRYTYRPSVPAHRRVRESPLSSDAIFKQSH.

The sequence for BnaDGAT1-80 is: MGHHHHHHGMEILDSGGVTMPTENGGADLDTLRHRKPRSDSSNGLLPDSV

TVSDADVRDRVDSAVEDTQGKANLAGENEIRESGGEAGG.

The unshaded segments represent the N-terminal tags in each case.

**Supplementary Table 1: Analysis of circular dichroism spectra for BnaDGAT1 truncations.**

To determine the influence of ligand binding on secondary structure, DGAT1 truncations were analyzed using DICHROWEB either in the absence and presence of ligands. The helix and strand secondary structure elements recorded were split into regular and distorted classes, with the definition of four residues per helix and two residues per strand as distorted. Data has been analysed with CDSSTR using SP175 reference data set.

| **Sample** | **Helix_(regular)_** | **Helix_(distorted)_** | **Strand_(regular)_** | **Strand_(distorted)_** | **Turns** | **Unordered** |
| --- | --- | --- | --- | --- | --- | --- |
| DGAT1_1-113_ | 0.36 | 0.11 | 0.10 | 0.08 | 0.11 | 0.24 |
| DGAT1_1-113_  + Oleoyl-CoA | 0.49 | 0.16 | 0.04 | 0.04 | 0.11 | 0.16 |
| DGAT1_1-113_  + CoA | 0.44 | 0.14 | 0.04 | 0.05 | 0.12 | 0.21 |
| DGAT1_1-80_ | 0.38 | 0.11 | 0.08 | 0.07 | 0.11 | 0.24 |
| DGAT1_1-80_  + Oleoyl-CoA | 0.14 | 0.06 | 0.15 | 0.12 | 0.14 | 0.39 |
| DGAT1_1-80_  + CoA | 0.45 | 0.15 | 0.05 | 0.06 | 0.11 | 0.19 |


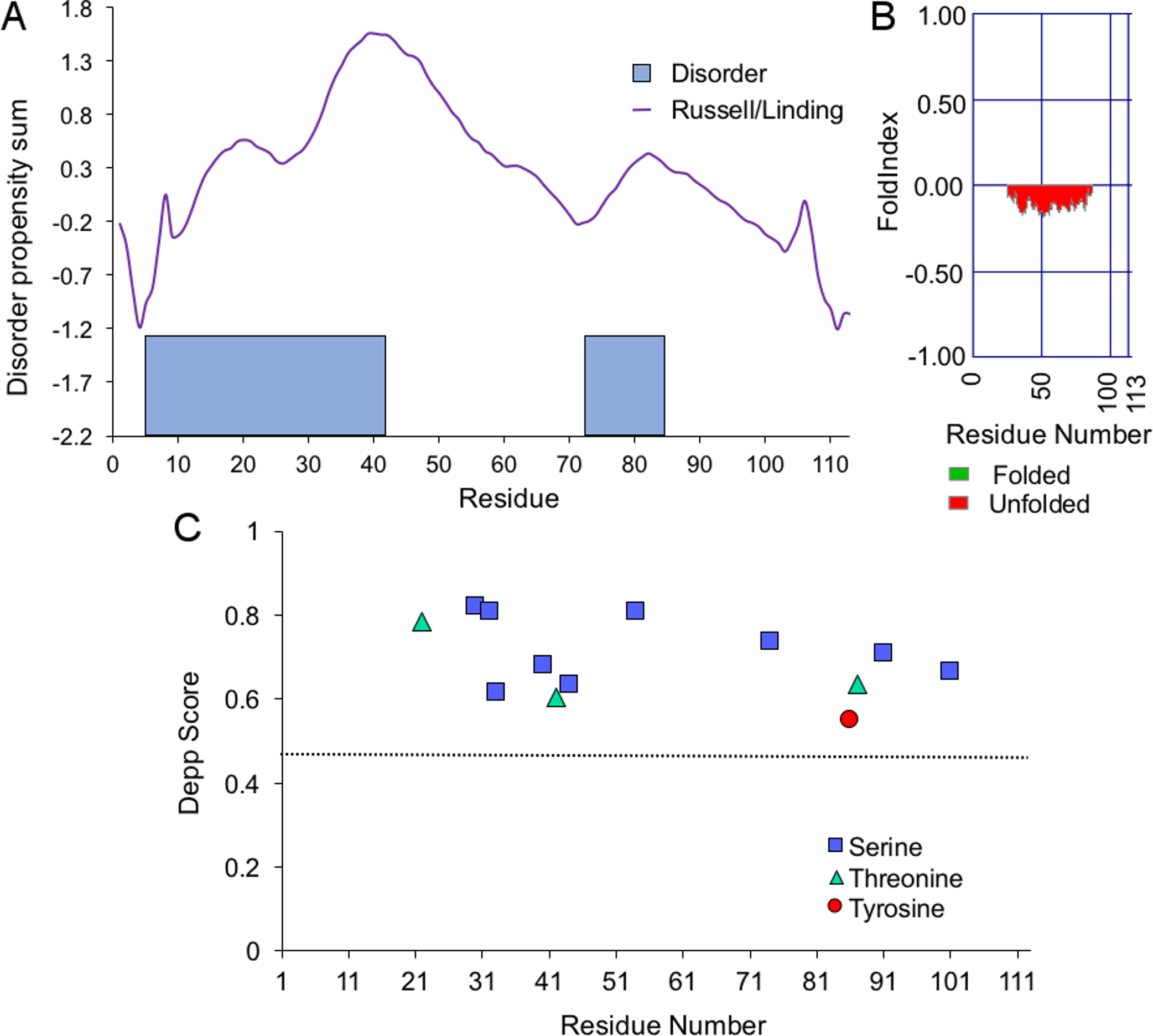


**Supplementary Figure 1.**

Bioinformatic analysis of N-terminal domain of BnaDGAT1. Disorder propensity calculated from **A**. GlobPlot. The region with high propensity of disorder is shown by blue box. **B**. FoldIndex. The disordered nature of the domain is shown by red shaded region. **C**. Prediction for potential phosphorylation sites. The residues having the higher propensity of phosphorylation are shown.

**
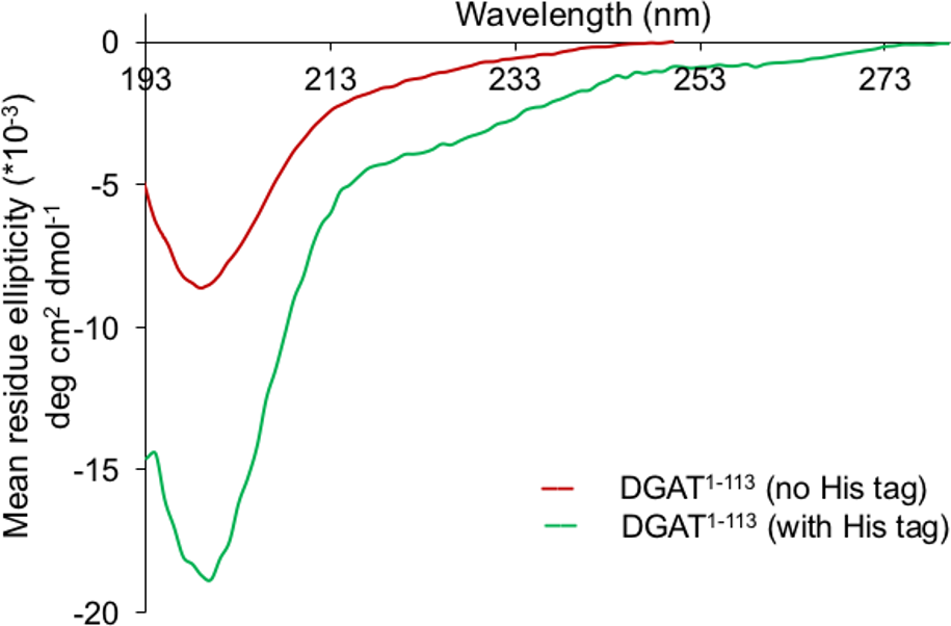
**

**Supplementary Figure 2.**

Circular dichroism (CD) profiles of various BnaDGAT1_1-113_ constructs with and without histidine tag.


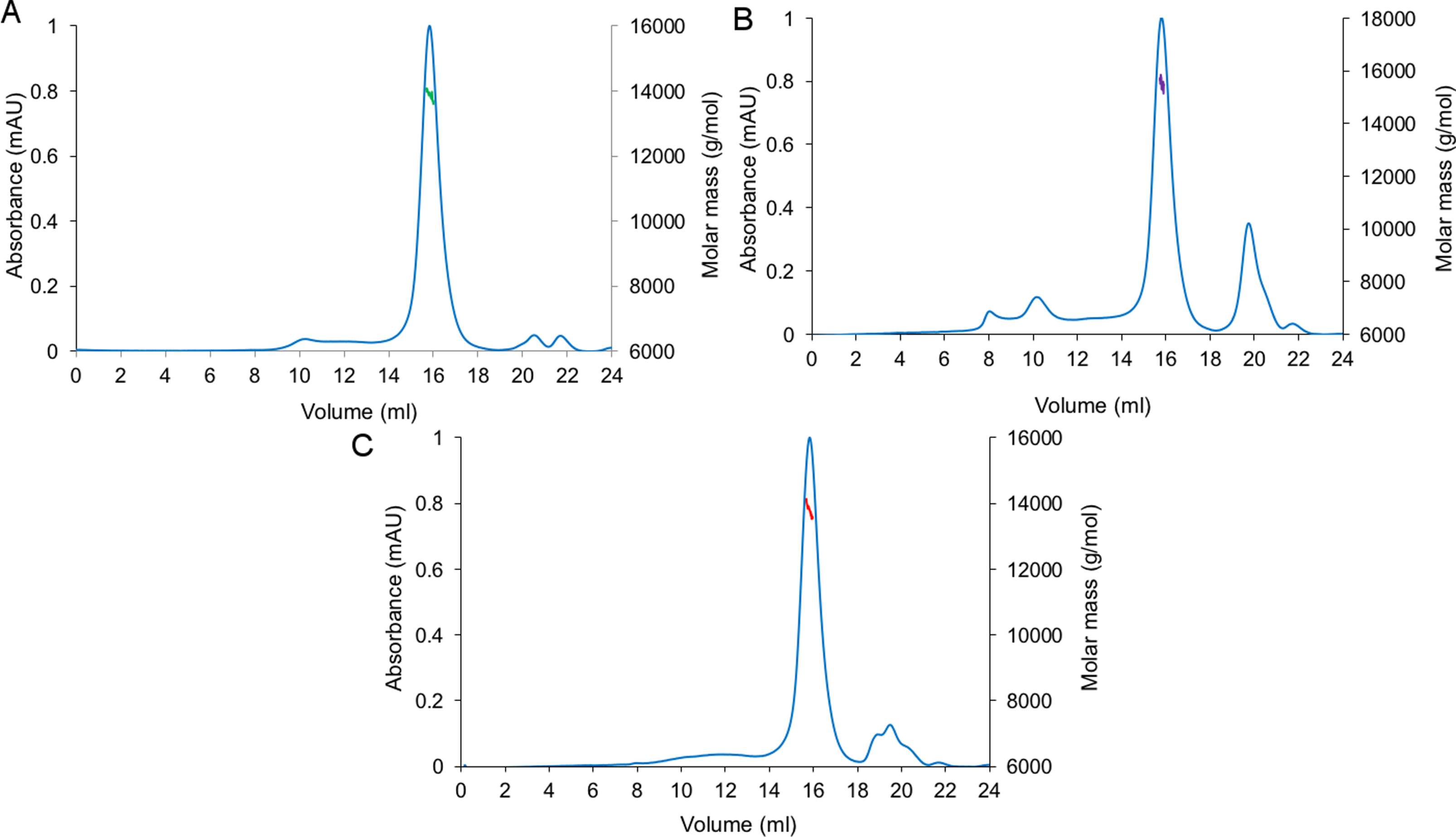


**Supplementary Figure 3.**

Analysis of BnaDGAT1_1-113_ oligomerization by SEC-MALLS **A.** apo form B. bound to oleoyl CoA and **C.** bound to CoA. The solid lines trace the absorbance at 280 nm of the eluate from a Superdex 200 10/300 column as a function of volume; the dotted lines represent the weight-average molecular weight of the species calculated from refractive index and light-scattering measurements.


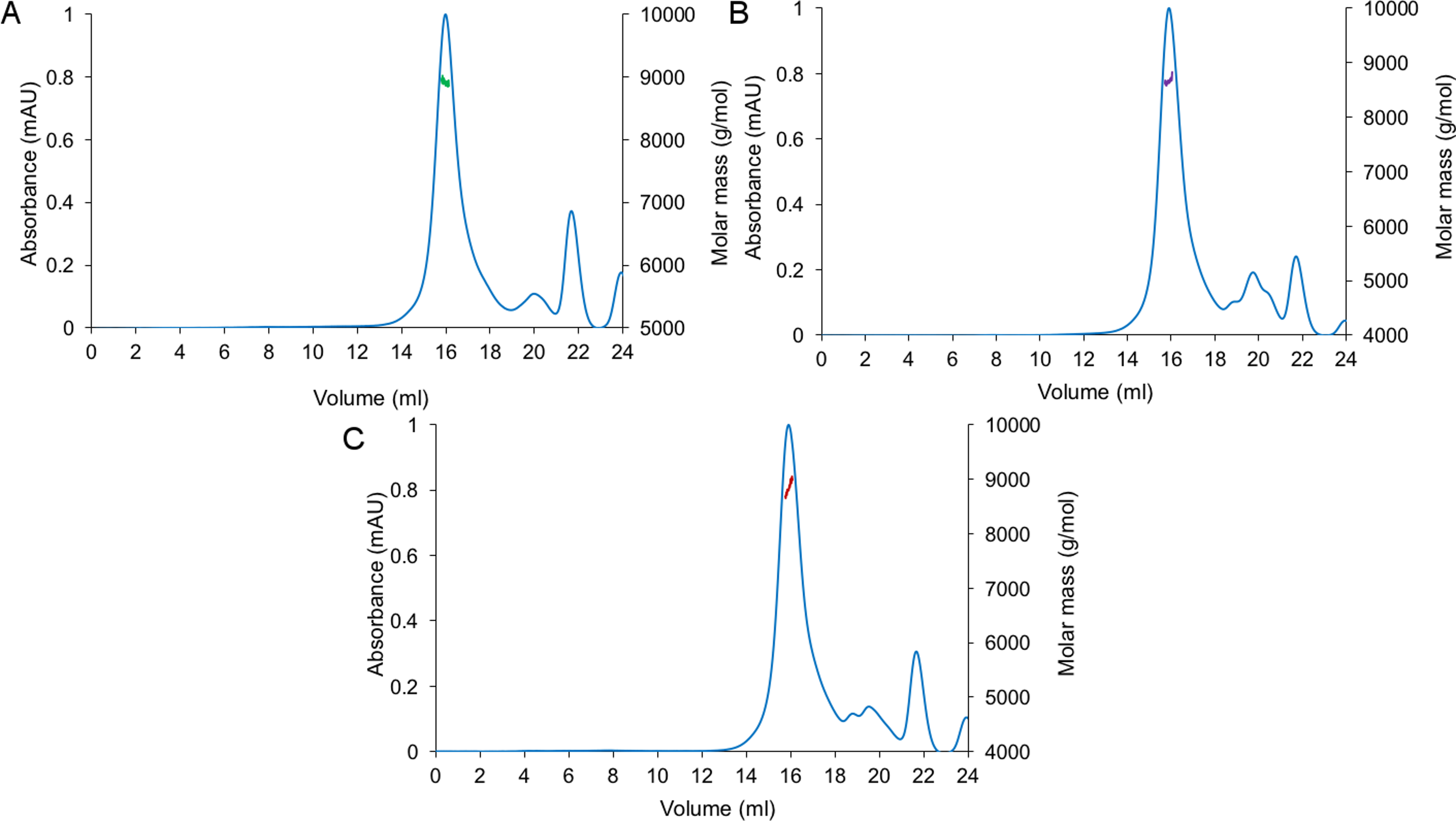


**Supplementary Figure 4.**

Analysis of BnaDGAT1_1-80_ oligomerization by SEC-MALLS **A.** apo form B. bound to oleoyl-CoA and **C.** bound to CoA The solid lines trace the absorbance at 280 nm of the eluate from a Superdex 200 10/300 column as a function of volume; the dotted lines represent the weight-average molecular weight of the species calculated from refractive index and light-scattering measurements


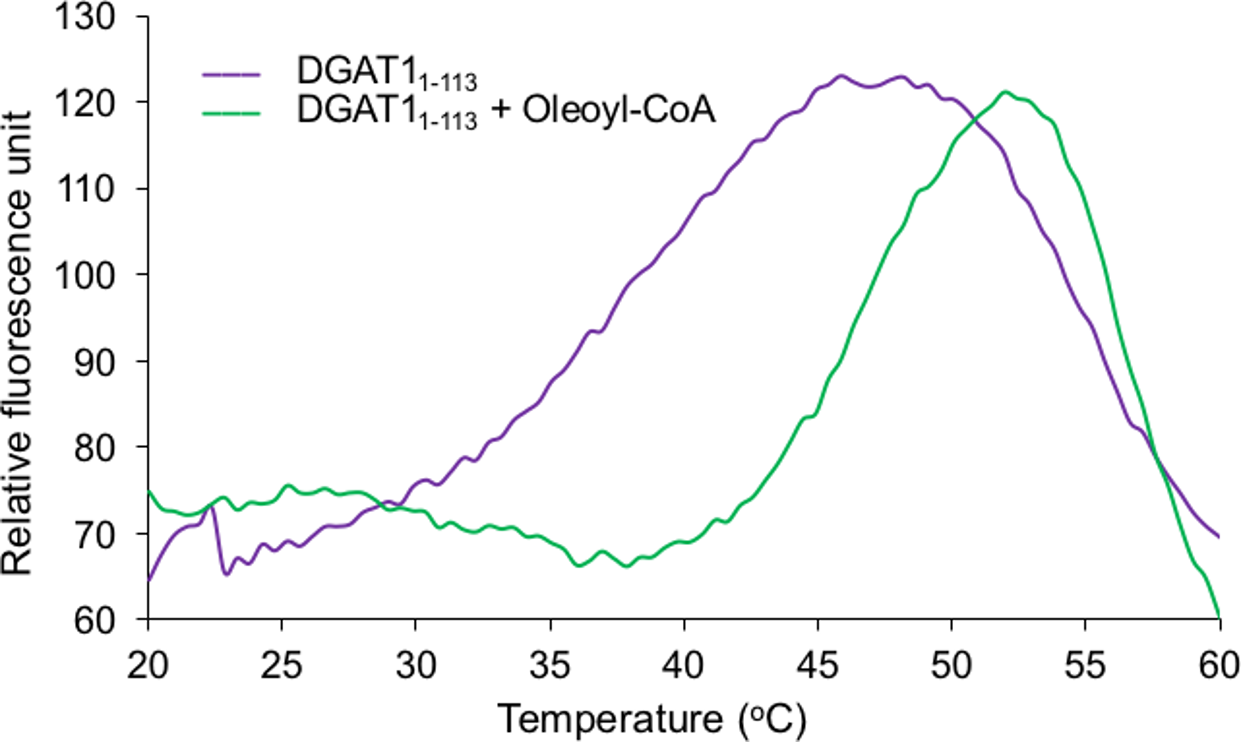


**Supplemental Figure 5.**

Thermal shift assay for BnaDGAT1_1-113_ and its oleoyl-CoA bound form in 25 mM phosphate pH 7.5, 300 mM NaCl. This was the best buffer condition obtained from the thermal shift assay screen. Buffers with pH 3-6 and pH 9-10 showed severe aggregation. Relative fluorescence against temperature for unbound protein is represented in magenta and bound form in green.

**
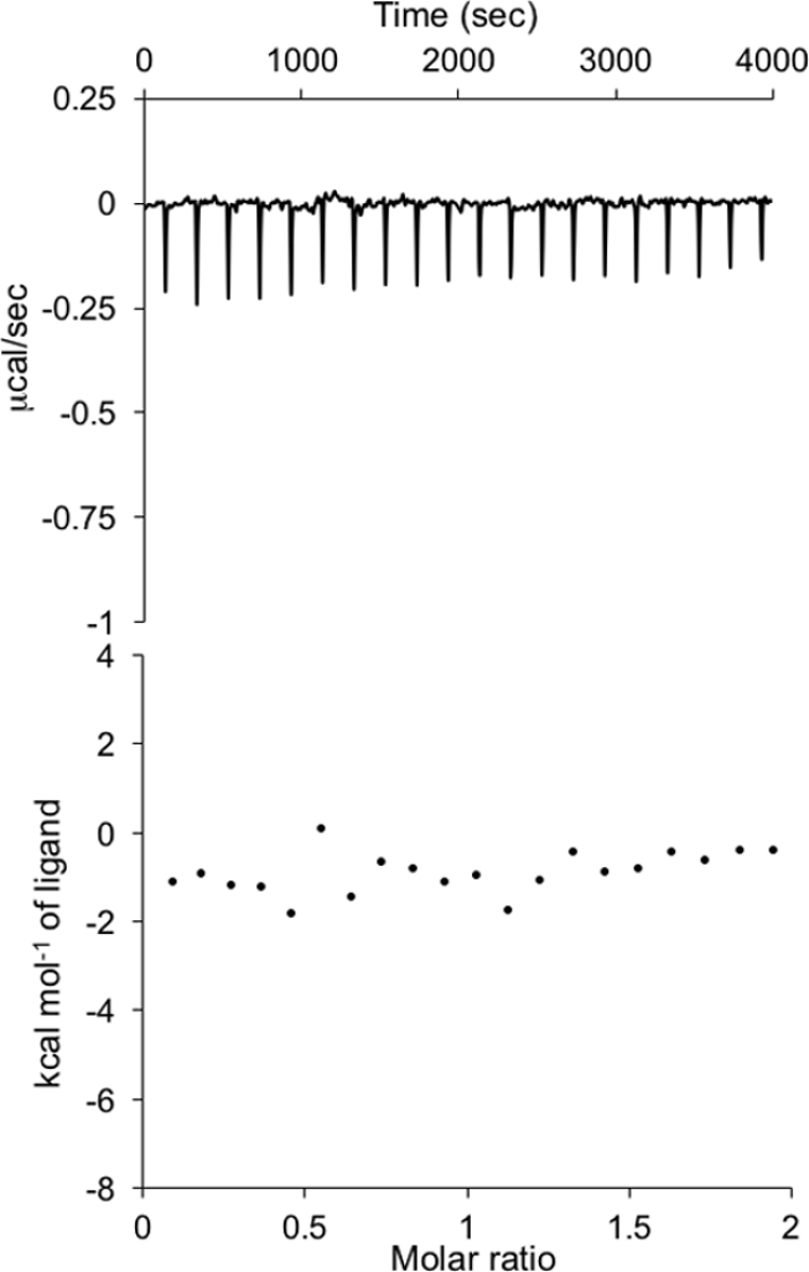
**

**Supplementary Figure 6.**

Isothermal titration calorimetric isotherms of protein BnaDGAT1_1-113_ titrated into buffer.

**
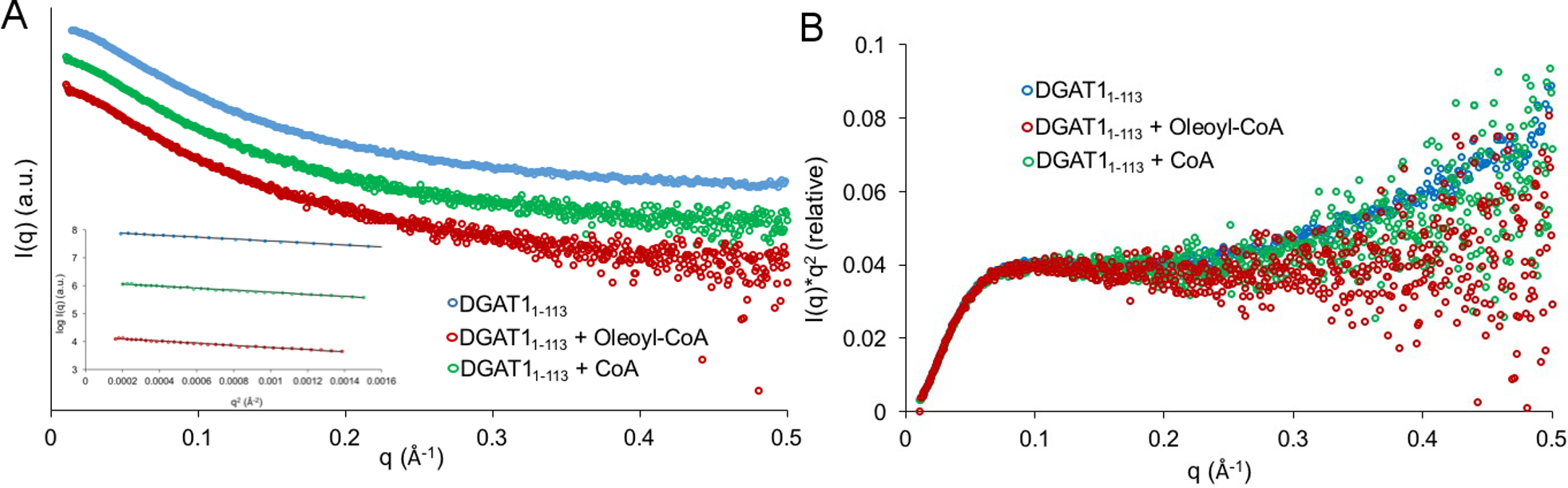
**

**Supplementary Figure 7.**

The SEC-SAXS profiles traces for all samples of BnaDGAT1_1-113_ for which the SAXS data was collected. R_g_(magenta), I_0_ (blue) and *I_qmin_* (*green*), which is an experimental intensity at very low q (q*Rg*<<1.0), are shown for each sample A. BnaDGAT1-113; **B**. BnaDGAT1_1-113_ + oleoyl-CoA; **C**. BnaDGAT1_1-113_ + CoA.


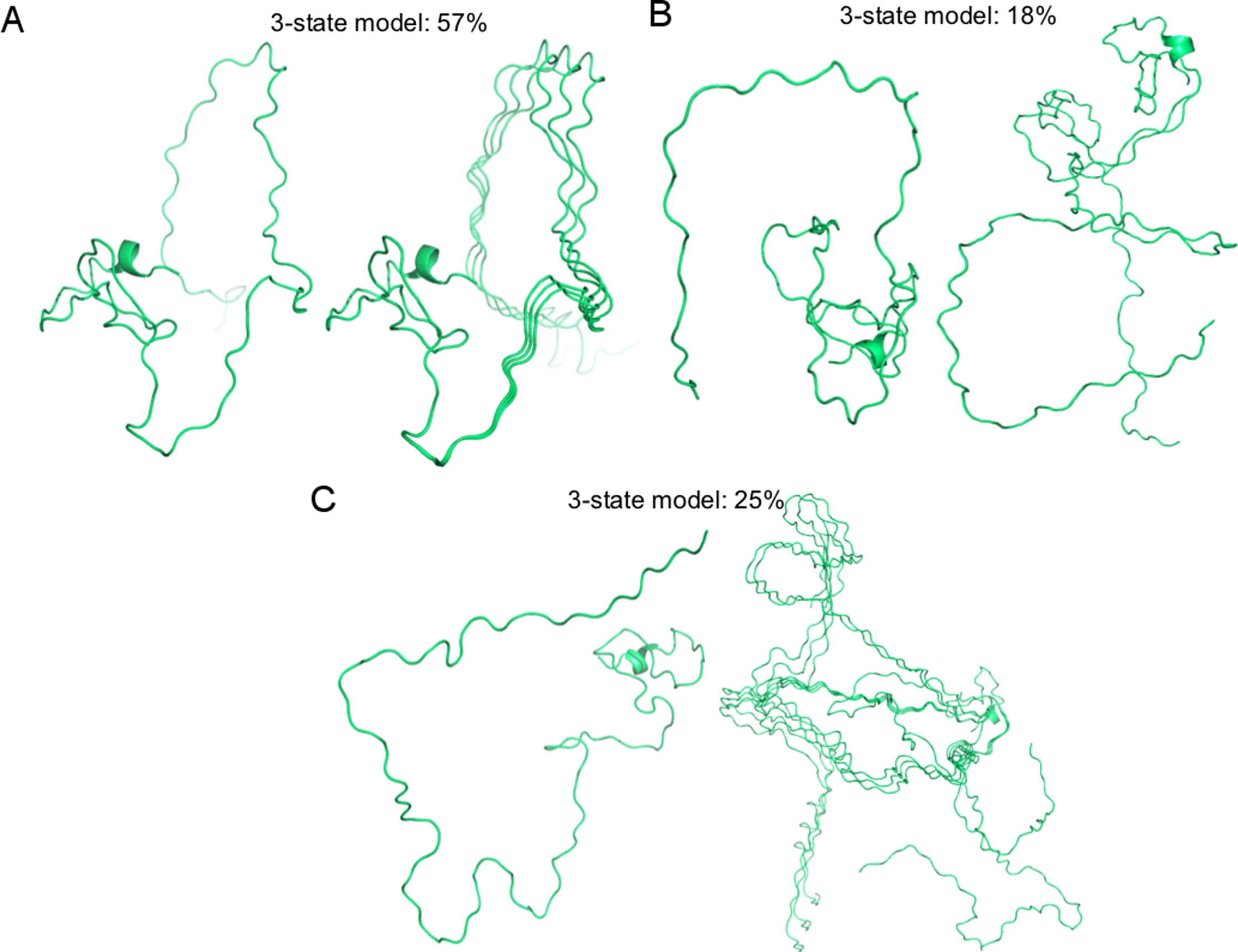


**Supplementary Figure 8.**

Conformations of the 3-state models for BnaDGAT1_1-113_ from SAXS analysis. In each case, the best scoring model is shown on the left and the top 10 combinations (some of them are duplicated) are aligned on the right. The ratio of population is also indicated.


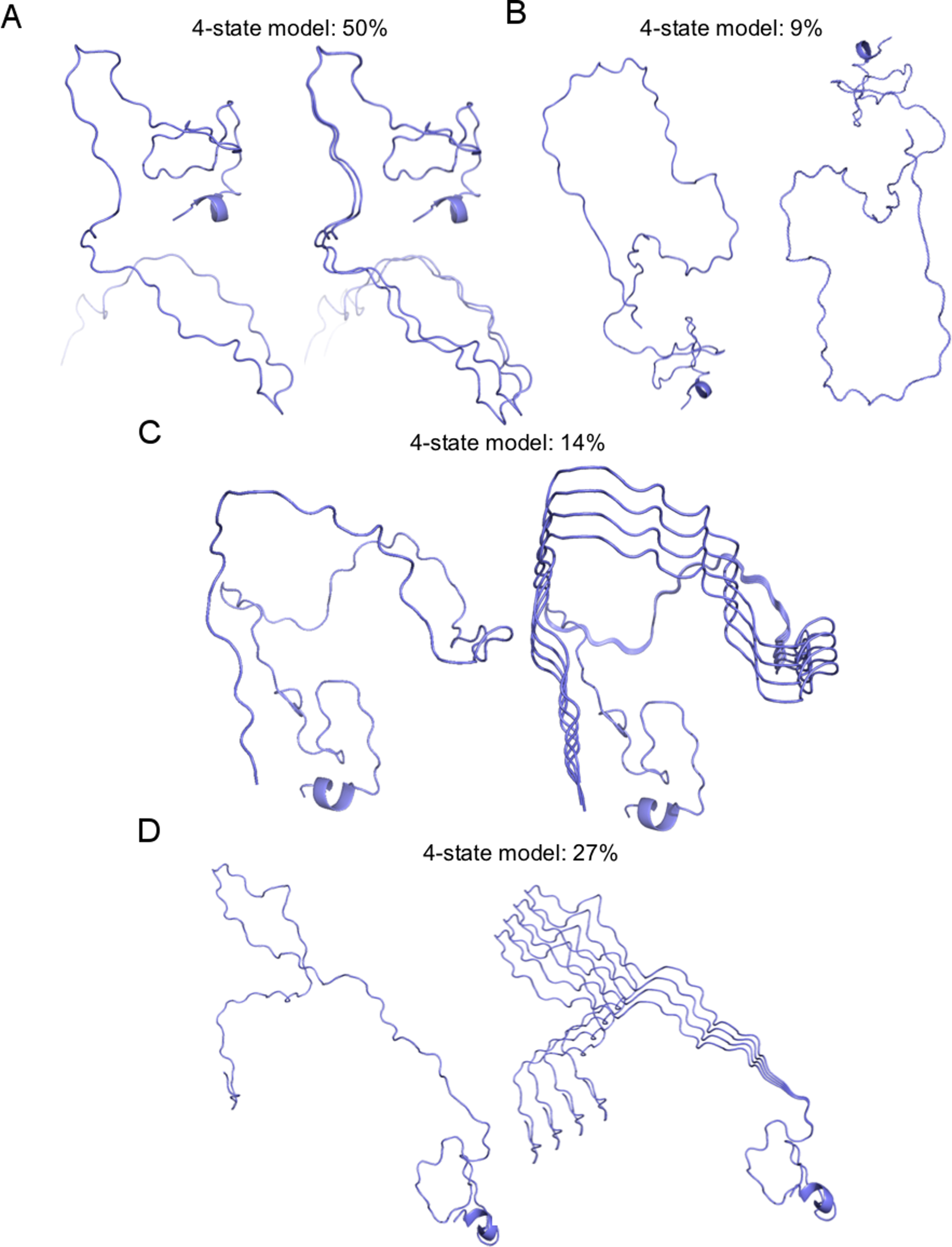


**Supplementary Figure 9.**

Conformations of the 4-state models for BnaDGAT1_1-113_ from SAXS analysis for 4-state models are represented. In each case, the best scoring model is shown on the left and the top 10 combinations (some of them are duplicated) are aligned on the right. The ratio of population is also indicated.


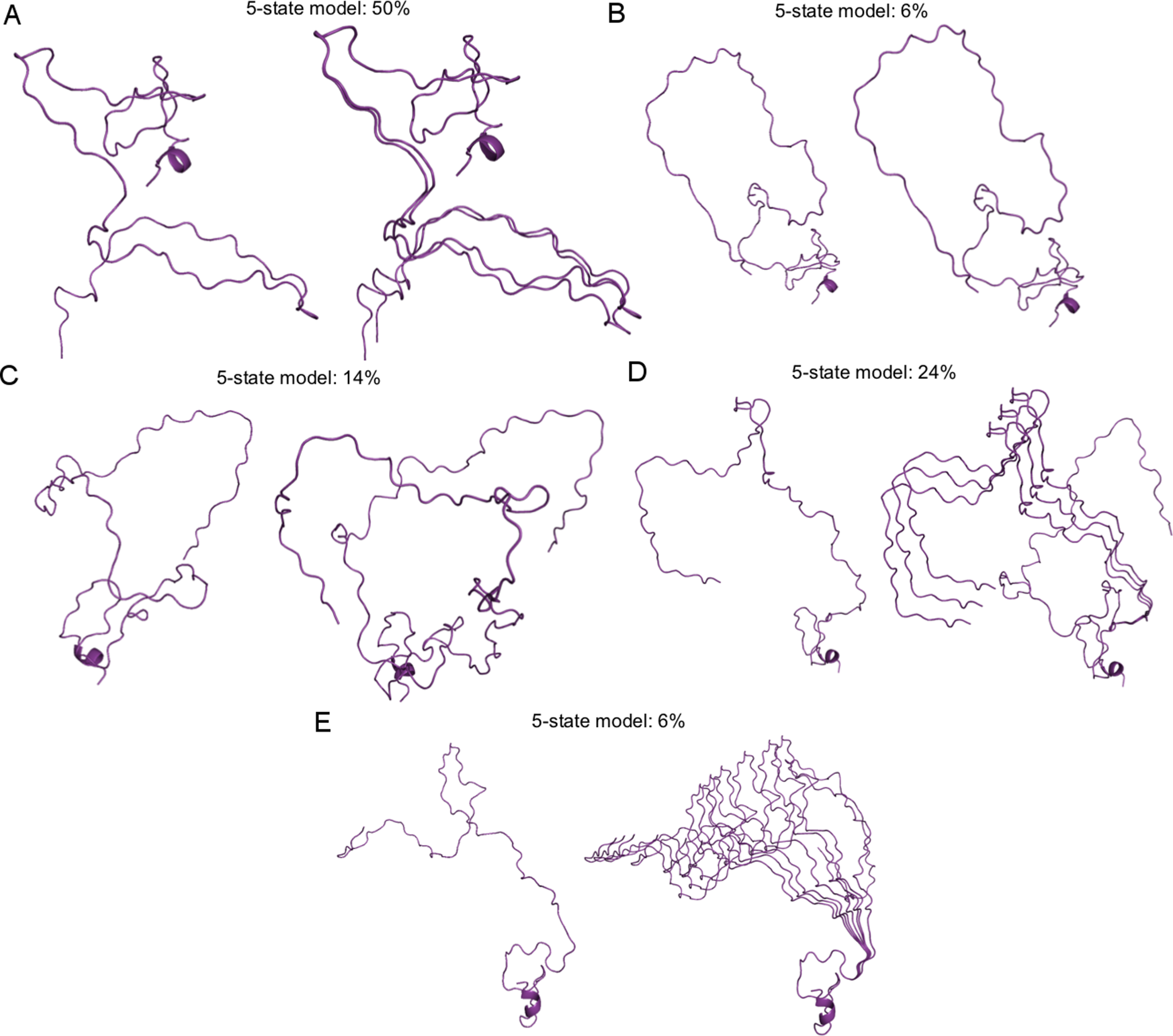


**Supplementary Figure 10.**

Conformations of the 5-state models for BnaDGAT1_1-113_ from SAXS analysis for 5-state models are represented. In each case, the best scoring model is shown on the left and the top 10 combinations (some of them are duplicated) are aligned on the right. The ratio of population is also indicated.
